# Melioidosis in India: A systematic review of individual cases

**DOI:** 10.1016/j.ijregi.2026.100843

**Published:** 2026-01-12

**Authors:** Nitin Gupta, Tirlangi Praveen Kumar, Astha Sethi, Pooja Kumari, Harpreet Kaur, Chiranjay Mukhopadhyay

**Affiliations:** 1 Department of Infectious Diseases, Kasturba Medical College, Manipal Academy of Higher Education, Manipal, India; 2 Division of Communicable Diseases, Indian Council of Medical Research, New Delhi, India; 3 Department of Microbiology, Kasturba Medical College, Manipal Academy of Higher Education, Manipal, India

**Keywords:** Melioidosis, India, Burkholderia pseudomallei

## Abstract

**Objectives:**

Melioidosis is increasingly recognized in India, but available evidence remains fragmented across small reports. We aimed to synthesize national case-based data to describe the clinical spectrum and identify factors associated with mortality.

**Methods:**

We systematically searched PubMed, Embase, and Web of Science from inception to February 5, 2025. Case reports, case series, and observational cohorts with individual patient-level culture- or molecular-confirmed data from India were included. Two reviewers independently screened studies, extracted data using a standardized form, and assessed quality using the Joanna Briggs Institute checklist for case reports. Univariate comparisons were performed between survivors and non-survivors. The protocol was registered on PROSPERO (CRD42025640317), and PRISMA guidelines were followed.

**Results:**

From 1983 screened records, 209 studies reporting 558 cases from 20 states were included. The mean age was 44.8 years, and 73.4% had diabetes. Bacteremia occurred in 70.1%; pulmonary disease (33.2%), bone/joint involvement (35.1%), splenic abscesses (17.7%), and central nervous system disease (13.3%) were the most common conditions. Among 498 cases with reported outcomes, mortality was 19.9%. Malignancy, pulmonary and central nervous system involvement, and bacteremia were associated with death.

**Conclusions:**

In India, melioidosis is widespread and often severe, with substantial mortality. Earlier recognition and improved diagnostic access are needed to reduce preventable deaths.

## Highlights

• Melioidosis is increasingly reported across diverse regions in India.
• High mortality driven by bacteremia and severe organ involvement.
• Diabetes is the most common underlying risk factor in India.
• Bone/joint and splenic disease showed lower mortality risk.
• Need for surveillance, early diagnosis, and national reporting.

## Introduction

Melioidosis, caused by the environmental Gram-negative bacillus *Burkholderia pseudomallei*, is an emerging yet under-recognized infectious disease in India [1]. Although traditionally associated with Southeast Asia and northern Australia, South Asia is now recognized as a significant region of concern [2]. Global modeling estimates suggest approximately 165,000 cases of melioidosis annually, with South Asia accounting for 44% of the global burden due to the large populations residing in ecologically favorable zones [3]. In India, vulnerability is further intensified by the convergence of frequent environmental exposure, particularly among the country’s large agricultural workforce, which is regularly engaged in rice farming, soil contact, and surface-water activities, and the high prevalence of diabetes mellitus, the strongest global predisposing factor for melioidosis [4]. Infection typically occurs through percutaneous inoculation, inhalation of contaminated aerosols, or ingestion of contaminated water, placing exposed agricultural and rural communities at substantial risk [5]. Despite growing clinical awareness, melioidosis continues to be substantially underdiagnosed: its manifestations mimic other tropical infections, and *B. pseudomallei* is often misidentified as *Pseudomonas* spp. in routine laboratories, compounded by the limited sensitivity of blood culture [4]. As early Indian observations noted, reported cases likely represent only a fraction of the true burden.

Clinical presentations of melioidosis range from localized abscesses to fulminant septicemia with multiorgan involvement [5,6]. Although diabetes is the predominant risk factor, conditions such as chronic kidney disease, hazardous alcohol use, and immunosuppressive conditions may further influence clinical expression and outcomes [[7], [8], [9]]. However, in India, the available literature remains fragmented, predominantly comprising isolated case reports and small institutional series [1]. This has limited the ability to characterize national patterns of organ involvement, antimicrobial management, and patient outcomes. Mortality in endemic regions can exceed 30% even with appropriate therapy [8], highlighting the importance of identifying clinical and host factors associated with a poor prognosis. To address these gaps, this systematic review aimed to synthesize all published case-based data on melioidosis from India, describe the demographic and clinical characteristics of reported patients, and identify factors associated with mortality.

## Methodology

### Objective and registration

The objective of this systematic review was to determine the proportion of melioidosis cases in India with poor outcomes, defined exclusively as reported mortality, and to identify clinical and demographic factors associated with death. The review protocol was prospectively registered on PROSPERO (CRD42025640317), and the study was conducted in accordance with the PRISMA guidelines (PRISMA checklist attached in supplementary file).

### Eligibility criteria

Studies were considered eligible if they reported individual case-level details of patients with melioidosis originating in India and included data on clinical presentation, treatment, and outcomes. Case reports, case series, and observational studies (both prospective and retrospective) were included in this review. Studies were excluded if they focused on non-human subjects, environmental isolates, or *in vitro* experiments. Similarly, studies that developed or validated diagnostic tests without individual clinical data, relapse or recurrence cohorts, or epidemiological studies lacking individual-level information were excluded. Reviews, editorials, commentaries, and conference abstracts were also not eligible. Only cases confirmed by culture or molecular methods were included; those diagnosed solely on clinical or serological grounds were excluded.

### Information sources and search strategy

A systematic search was conducted in PubMed, Embase, and Web of Science, covering studies published up to February 5, 2025. The search strategy used a combination of disease-specific terms and geographic keywords for India, joined with Boolean operators to maximize sensitivity. The complete search string was: *(melioidosis OR Whitmore OR pseudoglanders OR “Vietnamese time bomb” OR pseudomallei) AND (India OR Andhra OR Arunachal OR Assam OR Bihar OR Chhattisgarh OR Goa OR Gujarat OR Haryana OR Himachal OR Jharkhand OR Karnataka OR Kerala OR Madhya OR Maharashtra OR Manipur OR Meghalaya OR Mizoram OR Nagaland OR Odisha OR Punjab OR Rajasthan OR Sikkim OR Tamil OR Telangana OR Tripura OR Uttar OR Uttarakhand OR Bengal OR Andaman OR Chandigarh OR Dadra OR Daman OR Lakshadweep OR Delhi OR Puducherry OR Ladakh OR Kashmir).* The reference lists of included studies were also screened to identify additional relevant reports.

### Data extraction

Data were extracted using a standardized form based on a predefined coding dictionary. Study-level information included the first author’s last name, year of publication, and geographical location, with each patient assigned a case number. Patient-level variables captured were demographic details, comorbidities, and other reported risk factors. The clinical features, including the duration of illness at presentation and the presence of bacteremia confirmed by blood culture, were extracted. Organ system involvement was recorded when supported by clinical or imaging evidence and coded as pulmonary (pneumonia, infiltrates, or mediastinal nodes), pleural effusion, pericardial effusion or pericarditis, central nervous system (CNS) involvement (brain abscess or encephalitis), cutaneous involvement (abscess, ulcer, or cellulitis), hepatic abscess, splenic abscess, parotid infection, prostatic abscess, bone and joint involvement (osteomyelitis or septic arthritis), or cervical lymph node/neck abscess. Treatment data were classified into intensive and eradication phases. For the intensive phase, the primary intravenous antibiotic was coded as ceftazidime, meropenem/imipenem, or other agents. If both ceftazidime and a carbapenem were used, the drug administered for a longer duration was considered the primary agent. Intravenous therapy was categorized as either monotherapy or combination therapy with trimethoprim-sulfamethoxazole (TMP-SMX), and the duration was recorded in days. For the eradication phase, oral therapy was categorized as TMP-SMX or other agents (e.g., doxycycline, amoxicillin-clavulanate). In cases where >1 oral drug was used in combination with TMP-SMX, TMP-SMX was considered the primary eradication agent, consistent with established treatment guidelines. The primary outcome was patient mortality. Variables not reported in the original studies were coded as “not reported” and were not included in denominators.

### Data analysis

All extracted data were compiled in a database and analyzed. Descriptive statistics were generated to summarize patient characteristics. Mortality rates were calculated as proportions. To identify potential predictors of mortality, univariate analyses were performed to compare characteristics between survivors and non-survivors. Continuous variables were summarized as means and standard deviations (SDs) or medians and interquartile ranges (IQRs), as appropriate, and compared using independent t-tests or the Mann-Whitney U test. Categorical variables were summarized as frequencies and percentages and compared using χ² tests or Fisher’s exact tests, as applicable. A *P*-value <0.05 was considered statistically significant.

### Quality assessment

The methodological quality of all included case reports and case series was assessed using the Joanna Briggs Institute (JBI) critical appraisal checklists [10]. Because the objectives of this review focused on clinical characteristics, management, and mortality, the checklist item relating to adverse events was deemed not applicable and was therefore excluded from appraisal. Additionally, the items assessing clinical history and presenting features were combined into a single domain, as there was overlap in reporting across the included studies. Each case was evaluated for the clarity and completeness of demographic information, clinical presentation, diagnostic confirmation, treatment details, and follow-up. The critical appraisal was performed by two independent reviewers, with discrepancies resolved through consensus.

## Results

A total of 1983 records were identified through searches across three databases: Web of Science (n = 940), Embase (n = 628), and PubMed (n = 415) (Supplementary Figure 1). After removing 738 duplicate records, 1245 unique records remained for screening. Following title and abstract review, 889 records were excluded, leaving 356 articles for full-text assessment. Of these, seven reports could not be retrieved, and 349 full-text articles were assessed for eligibility. A further 140 reports were excluded for the following reasons: conference abstracts (n = 41), narrative reviews, commentaries, or letters (n = 36), studies conducted outside India (n = 15), animal or *in vitro* studies (n = 11), wrong pathogen (n = 2), relapse-focused cohorts (n = 1), epidemiological studies without individual case details (n = 1), absence of confirmatory diagnosis (n = 2), overlapping data (n = 3) and absence of clinical data (n = 1). Overall, 27 studies with 1754 cases were excluded because they lacked individual patient data. Ultimately, 209 studies fulfilled all eligibility criteria and were included in the final review (Supplementary Table 1).

### Baseline parameters

**Figure 1:**
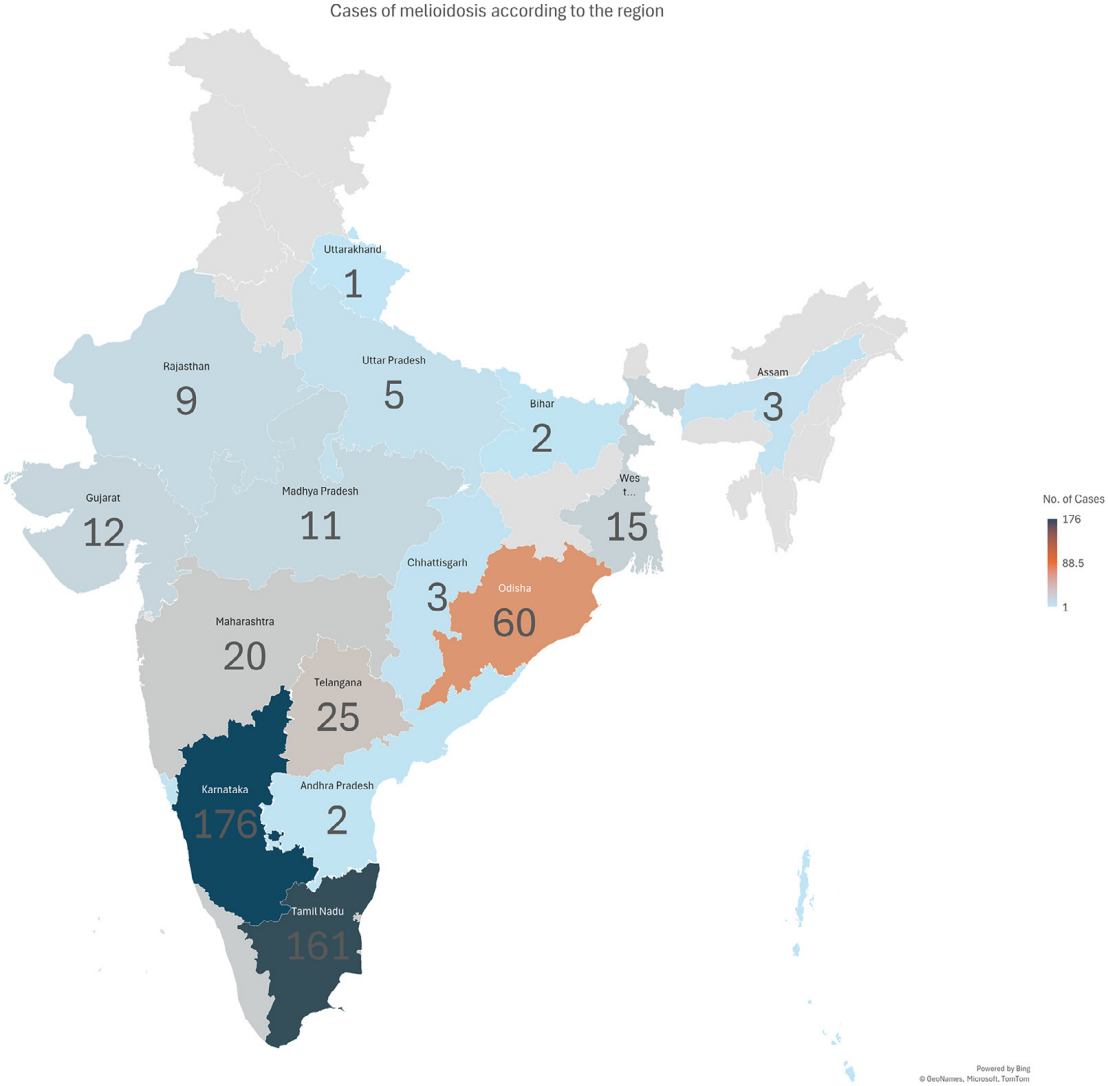
Geographic distribution of reported melioidosis cases in India by state and union territory.

A total of 558 confirmed cases of melioidosis were identified from published reports across 20 Indian states and union territories, spanning the period from 1991 to 2024 (Supplementary Table 1). The distribution of cases was markedly heterogeneous, with most concentrated in southern India. Karnataka (176 cases) and Tamil Nadu (161 cases) together accounted for over 60% of all reports, followed by Odisha (60 cases), Telangana (25 cases), and Pondicherry (22 cases) (Figure 1). Smaller clusters were noted in Maharashtra, Kerala, and West Bengal, while isolated cases were reported in central and northern states, including Madhya Pradesh, Gujarat, and Uttar Pradesh. Temporal analysis revealed a progressive increase in reported cases over time, particularly after 2008, reflecting improved clinical recognition, diagnostic capacity, and reporting from multiple tertiary centers across India (Supplementary Figure 2).

The mean age was 44.8 years (SD 14.8; *n* = 557). Six cases (1.1%) occurred in infants <1 year, 14 (2.5%) in children aged 1-15 years, 382 (68.6%) in middle-aged adults, and 155 (27.8%) in older people. Men accounted for 445 of 558 (79.7%) cases. Among reported comorbidities, diabetes mellitus was present in 356/485 patients (73.4%), HIV infection in 4/482 (0.8%), chronic kidney disease in 28/487 (5.7%), malignancy in 11/478 (2.3%), chronic lung disease in 20/465 (4.3%), and steroid use in 15/485 (3.1%).

Organ involvement was diverse: pulmonary disease in 185/558 (33.2%), pleural involvement 41/558 (7.3%), pericardial disease 7/558 (1.3%), CNS disease 74/558 (13.3%), cutaneous involvement 107/558 (19.2%), hepatic abscesses 70/558 (12.5%), splenic abscesses 99/558 (17.7%), parotid involvement 13/558 (2.3%), prostatic disease 33/558 (5.9%), bone or joint infection 196/558 (35.1%), cervical lymphadenitis 21/558 (3.8%), and vascular involvement 4/558 (0.7%). Blood culture positivity was reported in 288 of 411 (70.1%) cases.

Regarding treatment, in the intensive phase, ceftazidime was used in 268/480 patients (55.8%), carbapenems in 179/480 (37.3%), and other regimens in 33/480 (6.9%). Cotrimoxazole was given along with intensive therapy in 121/480 (25.2%). For eradication therapy, cotrimoxazole was the predominant agent used in 323/348 cases (92.8%), while doxycycline monotherapy was used in 21/348 (6.0%) and amoxicillin-clavulanate in 3/348 (0.9%). The median duration of intensive therapy was 14 days (IQR 14-21; *n* = 329), and the median duration of eradication therapy was 140 days (IQR 90-180; *n* = 258). Outcomes were available for 498 patients, of whom 99 (19.9%) died, and 399 (80.1%) survived.

### Factors associated with mortality

Male gender was less common among those who died (72.7% vs 82.2%, *P* = 0.034) (Table 1). Among comorbidities, malignancy was the only condition significantly associated with mortality (7.1% vs 1.0%, *P* < 0.001). At the same time, diabetes mellitus, chronic kidney disease, chronic lung disease, steroid use, and HIV infection showed no meaningful differences between groups. Clinical features associated with higher mortality included pulmonary involvement (51.5% vs 27.8%, *P* < 0.001), CNS involvement (23.2% vs 11.8%, *P* = 0.003), and bacteremia (77.8% vs 45.6%, *P* < 0.001). In contrast, splenic abscesses (9.1% vs 20.1%, *P* = 0.011) and bone or joint involvement (20.2% vs 39.3%, *P* < 0.001) were more common among survivors, suggesting an association with more localized disease. Regarding treatment, the choice of intensive-phase antibiotic did not differ significantly between groups: carbapenem use (34.4% vs 34.8%, *P* = 0.947) and ceftazidime use (65.6% vs 65.2%, *P* = 0.947) were nearly identical in both cohorts. Similarly, intensive-phase cotrimoxazole use (20.8% vs 24.7%, *P* = 0.466) and eradication-phase cotrimoxazole use (93.3% vs 92.8%, *P* = 0.937) were not associated with mortality.

**Table 1:** Comparison of chief demographic, comorbidity, clinical, and treatment variables between melioidosis patients who died (n = 99) and those who survived (n = 399).

Variable	Death (n = 99)	Survival (n = 399)	*P*-value
Male Gender	72/99 (72.7%)	328/399 (82.2%)	<0.001
Diabetes mellitus	63/99 (63.6%)	248/399 (62.2%)	0.716
Chronic kidney disease	9/99 (9.1%)	17/399 (4.3%)	0.080
Chronic lung disease	3/99 (3.0%)	14/399 (3.5%)	0.751
Steroid use	2/99 (2.0%)	12/399 (3.0%)	0.547
HIV	2/99 (2.0%)	1/399 (0.3%)	0.050
Malignancy	7/99 (7.1%)	4/399 (1.0%)	<0.001
Pulmonary	51/99 (51.5%)	111/399 (27.8%)	<0.001
Central nervous system	23/99 (23.2%)	47/399 (11.8%)	0.003
Spleen	9/99 (9.1%)	80/399 (20.1%)	0.011
Bone/Joint	20/99 (20.2%)	157/399 (39.3%)	<0.001
Bacteremia	77/99 (77.8%)	182/399 (45.6%)	<0.001
Choice of IP - carbapenem	22/64 (34.4%)	126/362 (34.8%)	0.947
Choice of IP - ceftazidime	42/64 (65.6%)	236/362 (65.2%)	
IP with cotrimoxazole	16/77 (20.8%)	94/381 (24.7%)	0.466
Choice of EP - cotrimoxazole	14/15 (93.3%)	296/319 (92.8%)	0.937

CKD, Chronic kidney disease; CNS, Central nervous system; HIV, Human immunodeficiency virus; IP, Intensive phase.

### Critical appraisal of the included studies

Overall reporting quality across the 558 included cases was variable but generally adequate for extracting core clinical variables (Supplementary Table 2). Demographic details were clearly described in 484 cases (86.7%), while clinical history and presentation were sufficiently documented in 459 cases (82.3%). Diagnostic confirmation using culture or molecular methods met JBI standards in 413 cases (74.0%), although several reports lacked explicit details on sample type or laboratory methods. Treatment information was the least consistently reported domain, available in 326 cases (58.4%), reflecting frequent omissions regarding antibiotic choice, dosage, or duration. Follow-up and outcomes, including survival status, were reported in 498 cases (89.2%), though the length of follow-up was rarely specified. The exclusion of the adverse-event domain did not affect the appraisal, as none of the reports were designed to evaluate the safety of the intervention. Overall, the critical appraisal highlights reasonable completeness in core clinical data but identifies essential gaps in treatment reporting and diagnostic detail.

## Discussion

This systematic review identified 558 confirmed cases of melioidosis reported from 209 studies across 20 Indian states over 3 decades, with a marked rise in publications after 2008 and a concentration of cases in southern India, particularly Karnataka and Tamil Nadu. The demographic profile mirrored global patterns, with a predominance of middle-aged men and a high prevalence of diabetes. Clinical presentations were diverse, spanning pulmonary, neurological, musculoskeletal, splenic, hepatic, prostatic, and cutaneous involvement, while bloodstream infection occurred in more than two-thirds of cases. Treatment practices varied substantially, although ceftazidime and carbapenems remained the most frequently used intensive-phase agents, and cotrimoxazole was almost universally employed for eradication therapy. Overall mortality was 19.9%, with deaths primarily associated with bacteremia, pulmonary and CNS involvement, and underlying malignancy, while localized abscess-predominant disease, particularly splenic and bone/joint involvement, was associated with survival. Collectively, these findings highlight substantial geographical heterogeneity, a broad clinical spectrum, and persistent mortality in melioidosis cases reported from India.

A striking feature of the available literature is the marked regional heterogeneity in reported melioidosis cases across India, with a disproportionate concentration from southern coastal states such as Karnataka and Tamil Nadu. Environmental factors such as high rainfall, tropical humidity, and warm temperatures create favorable conditions for *B. pseudomallei* survival and transmission in soil and water [11,12]. These climatic features are comparable to well-established endemic hotspots in Southeast Asia and northern Australia, suggesting that ecological suitability in coastal India may be substantially underestimated. Strengthening soil and environmental surveillance in high-rainfall districts may help identify additional concealed hotspots [12]. While this pattern may partly reflect true ecological suitability, it is also likely influenced by substantial reporting and diagnostic biases. Much of the Indian literature originates from tertiary-care centers with established microbiology services and heightened clinical awareness. In contrast, diagnostic capacity for melioidosis appears to be more variable in many regions of northern and central India. Consequently, the apparent geographic distribution in published reports should not be interpreted as a reliable indicator of the actual disease burden. Instead, it likely represents a composite of ecological risk, laboratory availability, referral pathways, and publication practices.

The demographic profile mirrors global trends, with melioidosis affecting >70% of cases, similar to the 70% prevalence in the Thailand cohort and higher than the 45% reported in Darwin [7,8]. Observational studies from India consistently show that >70% of the melioidosis patients have diabetes mellitus [[13], [14], [15], [16], [17], [18], [19], [20], [21], [22], [23], [24]]. Nonetheless, a noteworthy subset of Indian cases occurred in adults and children without diabetes mellitus. This highlights that exposure-driven disease can occur even in otherwise healthy individuals, particularly in agricultural or flood-affected settings. Increased clinical vigilance for melioidosis in febrile children presenting with abscesses or severe sepsis, especially during monsoon seasons, could support earlier diagnosis and management. Further research is needed to understand the distinct epidemiology of melioidosis in children, including potential host or environmental determinants.

The clinical spectrum of melioidosis in India is broad and heterogeneous. Pulmonary involvement, osteoarticular infection, splenic and hepatic abscesses, and cutaneous disease were common. In contrast, bloodstream infection occurred in approximately 70% of cases, which is intermediate between the rates reported in Darwin (56%) and Thailand (77.1%) [7,8]. A particularly striking finding is the high frequency of CNS involvement in the Indian data (13.3%), which far exceeds the rates reported in prospective cohorts from Darwin (<2%) and Thailand (0.8%). This discrepancy is likely reflected in multiple factors. Reporting bias is a significant contributor, as the Indian literature is dominated by case reports and small case series that tend to preferentially highlight rare or diagnostically challenging presentations, such as neuromelioidosis. This is supported by a relatively low incidence of neuromelioidosis (≤3%) in observational studies from India [[13], [14], [15], [16], [17], [18], [19], [20], [21], [22], [23], [24]]. Broader diagnostic definitions used in this review, including diagnosis based on cerebrospinal fluid pleocytosis combined with a culture from another site or characteristic magnetic resonance imaging findings, may further inflate the proportion of CNS disease compared with the stricter microbiological confirmation criteria applied in large prospective cohorts. Beyond these methodological explanations, emerging microbiological evidence suggests a potential biological basis for neurotropism in India. In a recent cohort from southern India, the BimABm allele, an established virulence factor associated with neural invasion, was detected in 5.8% of isolates and was strongly linked to neuromelioidosis [25].

Interestingly, the prevalence of prostatic involvement in this review was markedly lower than that reported from northern Australia, where prostate abscesses are a common manifestation of melioidosis and often drive the diagnostic pathway [7,26]. Several factors may contribute to this difference, including regional variation in *B. pseudomallei* strains, differences in host factors, under-recognition due to limited use of pelvic imaging in resource-constrained settings, or delayed referral patterns. The low detection rate of prostatic disease in Indian reports may therefore reflect a combination of true epidemiological variation and underdiagnosis. Importantly, prostatic melioidosis carries a meaningful risk of relapse if not identified and adequately drained, as antibiotic penetration into prostatic tissue is limited, and undrained abscesses can persist as reservoirs for recurrent infection [26]. Surgical or image-guided drainage is, therefore, a key component of management when prostate abscesses are present. The low apparent prevalence in Indian literature may thus reflect both epidemiological differences and missed diagnoses, underscoring the need for targeted imaging in men presenting with groin discomfort, urinary symptoms, or persistent fever despite appropriate therapy [27].

The mortality in Indian reports (19.9%) appears lower than that observed in highly endemic settings. In Darwin, acute in-hospital mortality was 12% overall, but was historically much higher before improvements in critical care, while the Thai multicenter cohort reported 24.9% mortality at 1 month and 33.9% at 1 year [7,8]. The mortality estimate in this systematic review should be interpreted with caution. Published case reports and series are inherently prone to reporting bias, with clinicians more likely to document survivors or unusual presentations than rapidly fatal cases. Moreover, septic melioidosis can progress fulminantly, and many patients may die before a microbiological diagnosis is established, leading to under-ascertainment of fatal outcomes. Mortality is also strongly influenced by the stage and nature of presentation; acute bacteremic disease carries a substantially higher risk of death than chronic or localized forms. Consistent with this variability, mortality rates reported in observational cohorts from India range widely, from 0% to 57%, with most series reporting rates between 3% and 39%, reflecting differences in case mix, diagnostic capacity, and clinical pathways [[13], [14], [15], [16], [17], [18], [19], [20], [21], [22], [23], [24]]. These factors, together, make a direct comparison with mortality rates from highly endemic regions challenging.

A notable finding in our analysis was the association of splenic abscesses and bone/joint involvement with survival. While counterintuitive, this pattern has been observed in other endemic settings and is likely a reflection of differences in pathophysiology [6]. Abscess-predominant disease often represents a more localized or subacute infection, arising from effective containment of *B. pseudomallei* within granulomatous or walled-off lesions. Such localization limits hematogenous dissemination and reduces the risk of overwhelming sepsis, which is the major driver of mortality. In contrast, bacteremia, pulmonary involvement, and CNS disease reflect systemic spread and high organism burden, which are hallmark predictors of poor outcomes. Thus, the presence of discrete abscesses, particularly in the spleen or musculoskeletal system, may paradoxically serve as a marker of robust host immune containment rather than severe disease. Recognition of these patterns is relevant for clinical prognostication and may help guide imaging and monitoring strategies.

Considerable heterogeneity was also observed in reported treatment practices across studies, particularly with respect to the choice of intensive-phase antimicrobials, the use of combination therapy, and the reporting of treatment duration. While ceftazidime and carbapenems were the most frequently used agents, the selection often appeared to be influenced by local availability, illness severity, and institutional protocols rather than standardized national guidance. Importantly, in our analysis, no significant difference in mortality was observed between patients treated with ceftazidime and those treated with carbapenems, consistent with prior randomized trial data [28]. Apparent treatment-related differences in outcomes reported in individual case series are therefore likely confounded by disease severity, timing of presentation, and selection bias inherent to case-based literature. Cotrimoxazole remained the predominant eradication therapy, consistent with international recommendations [29]. However, eradication-phase therapy was inconsistently documented, limiting any meaningful assessment of its impact on relapse or long-term outcomes. These findings underscore the need for standardized treatment pathways and prospective multicenter data to elucidate better how variations in antimicrobial management impact outcomes in the Indian context.

This review also highlights the persistent challenges in India’s diagnostic and healthcare systems. Misdiagnosis as tuberculosis, limited availability of culture facilities, and delayed microbiological confirmation were recurrent themes [4,[30], [31], [32], [33], [34]]. With the expanding geographic footprint of *B. pseudomallei* across India and the country’s substantial diabetes burden, melioidosis should now be recognized as an emerging public health concern. Improving laboratory capacity, promoting clinician awareness, ensuring access to appropriate antimicrobials, and developing standardized national management guidelines are urgently needed. Prospective multicenter studies incorporating molecular typing, severity scoring, and systematic long-term follow-up would be invaluable in clarifying disease burden, antimicrobial resistance patterns, and predictors of adverse outcomes in the Indian context.

Finally, several limitations inherent to this review must be acknowledged. The reliance on case reports and small series introduces publication and selection bias, and key variables such as treatment timing, organ-specific severity, and relapse rates were inconsistently reported. Mortality may be underestimated due to the preferential publication of survivors or diagnostically interesting presentations. Despite these limitations, this review integrates fragmented evidence. It provides important insights into the evolving epidemiology and clinical profile of melioidosis in India, underscoring the need for robust prospective surveillance and molecular epidemiology to understand better regional disease patterns, including the neurotropic potential of the disease.

## Conclusion

Melioidosis in India is geographically widespread, frequently severe, and associated with substantial mortality, particularly among patients with bacteremia, pulmonary involvement, CNS disease, or malignancy. Improved clinical recognition and early access to appropriate diagnostics and therapy are crucial to reducing preventable deaths. Strengthening national surveillance, making melioidosis a notifiable disease, and supporting its inclusion among neglected tropical diseases would enable better resource allocation, targeted prevention, and policy prioritization to address this emerging public health threat.

## Declaration of competing interest

The authors have no competing interests to declare.

## Appendix Supplementary materials

Image, application 1

Image, image 2 dummy alt text

Image, image 3 dummy alt text

Image, application 4

Image, application 5

Image, application 6
