# Supplementary figures and images for: Melioidosis in India: A systematic review of individual cases
Source: IJID Reg. 2026 Jan 12;18:100843. doi: 10.1016/j.ijregi.2026.100843 (PMC12874796; doi:10.1016/j.ijregi.2026.100843)

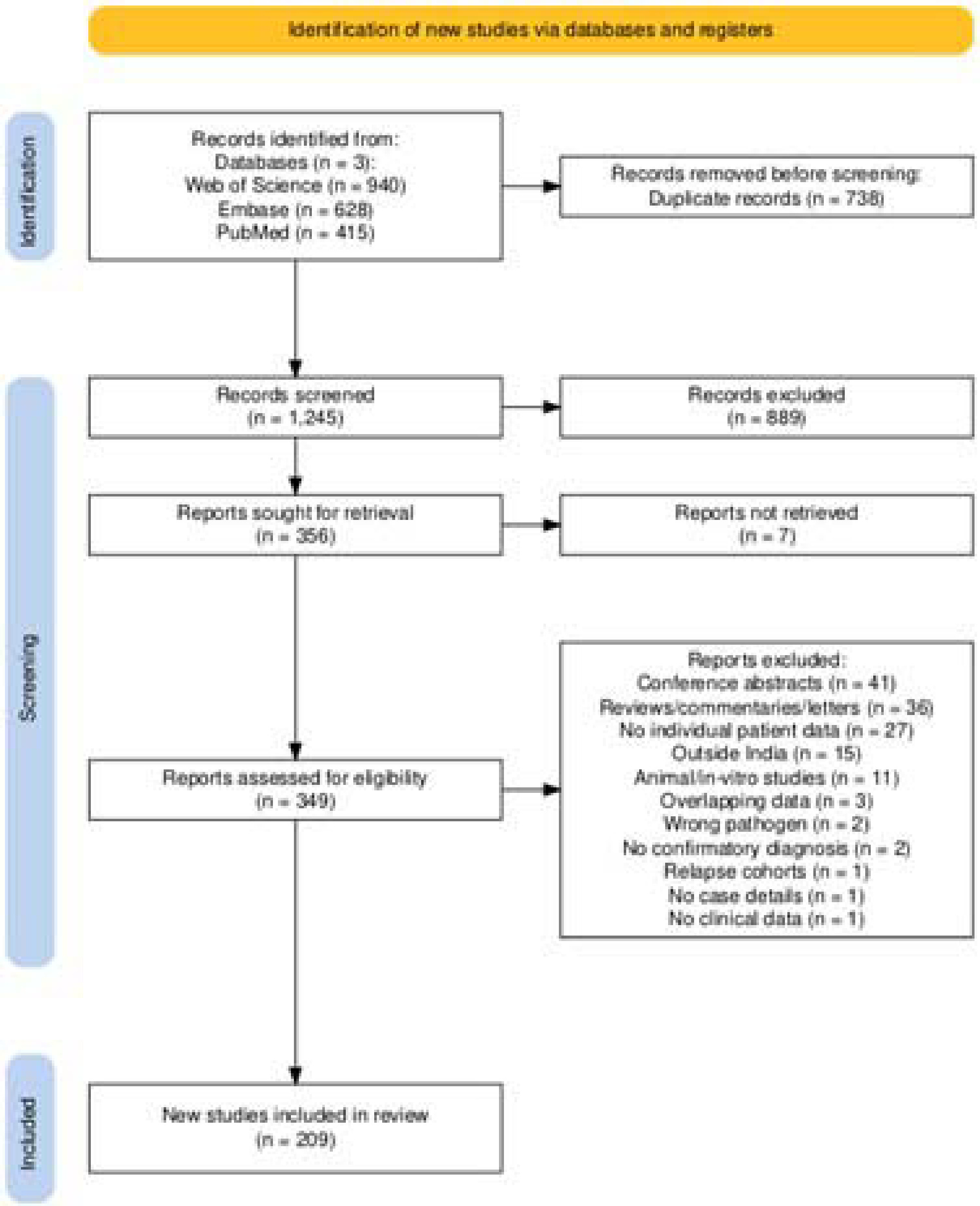

Supplement: Supplementary file 2 [file mmc2.jpg]

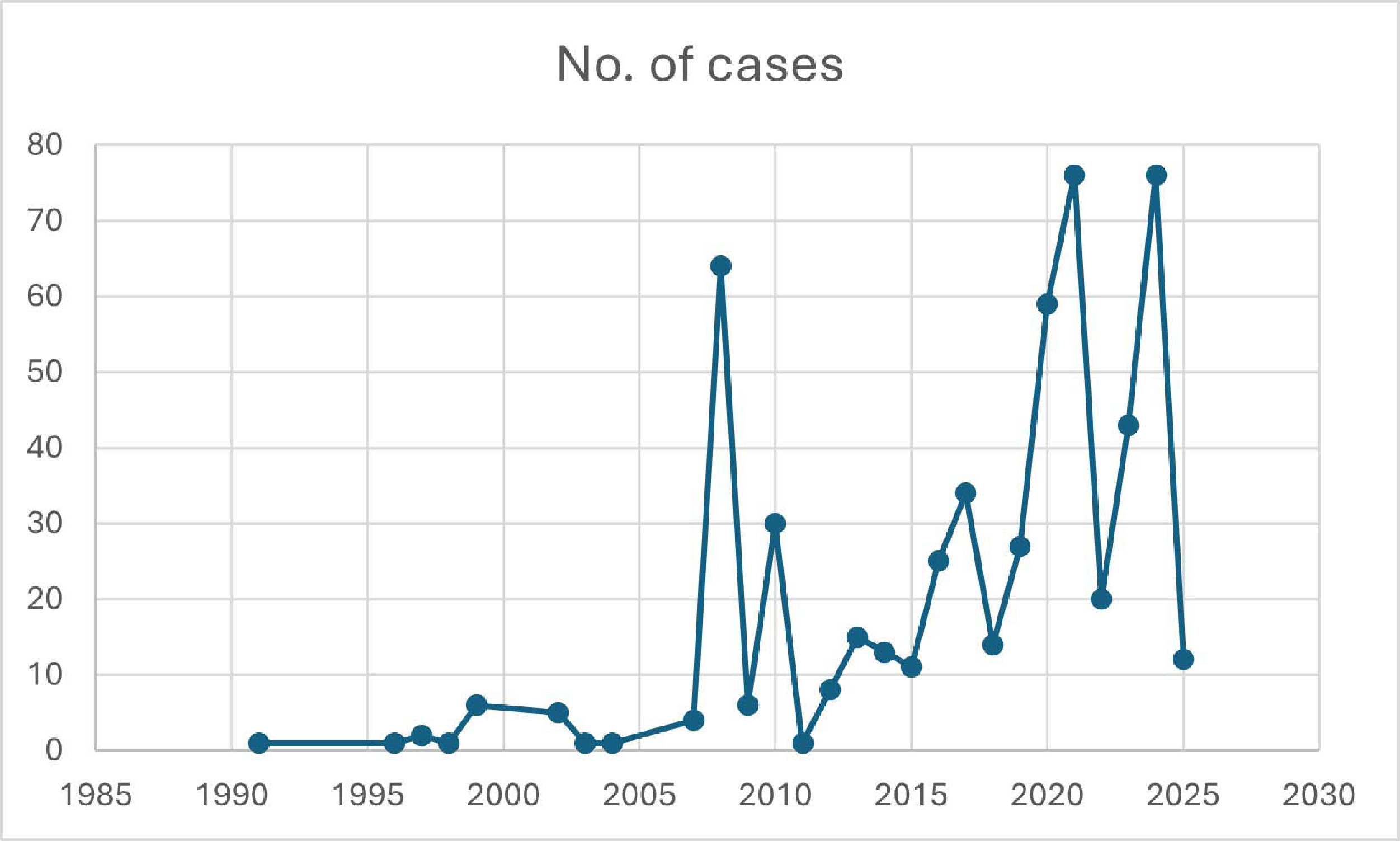

Supplement: Supplementary file 3 [file mmc3.jpg]
