# Supplementary material for: Melioidosis in India: A systematic review of individual cases
Source: IJID Reg. 2026 Jan 12;18:100843. doi: 10.1016/j.ijregi.2026.100843 (PMC12874796; doi:10.1016/j.ijregi.2026.100843)
Supplement: Supplementary file 4 [file mmc4.docx]

**Supplementary Materials**

- **Supplementary Table 1**: Details of all included studies with individual case details on melioidosis
- **Supplementary Table 2:** Joanna Briggs Institute critical appraisal results for included case reports
- **Supplementary Figure 1**: PRISMA diagram showing screening and inclusion of studies
- **Supplementary Figure 2**: Annual number of reported melioidosis cases in India with available clinical profile and outcome data (1991–2024)
- **PRISMA checklist**
