# Supplementary material for: Melioidosis in India: A systematic review of individual cases
Source: IJID Reg. 2026 Jan 12;18:100843. doi: 10.1016/j.ijregi.2026.100843 (PMC12874796; doi:10.1016/j.ijregi.2026.100843)
Supplement: Supplementary file 5 [file mmc5.docx]

Supplementary Table 1: Details of all included studies with individual case details on melioidosis

| **Sn** | | **Author** | | **State** | | **Year** | | **No. of cases** |
| --- | --- | --- | --- | --- | --- | --- | --- | --- |
| 1 | | Dash et al.[1] | | Odisha | | 2022 | | 2 |
| 2 | | Patil et al.[2] | | Maharashtra | | 2016 | | 3 |
| 3 | | Easow et al.[3] | | Pondicherry | | 2024 | | 5 |
| 4 | | Pande et al.[4] | | Tamil Nadu | | 2018 | | 1 |
| 5 | | Mohanty et al.[5] | | Odisha | | 2020 | | 9 |
| 6 | | Varma et al.[6] | | Kerala | | 2021 | | 1 |
| 7 | | Mamtora et al.[7] | | Maharashtra | | 2018 | | 1 |
| 8 | | Bojanapati et al.[8] | | Tamil Nadu | | 2024 | | 1 |
| 9 | | Singh et al.[9] | | Maharashtra | | 2015 | | 1 |
| 10 | | Yadav et al.[10] | | Madhya Pradesh | | 2023 | | 7 |
| 11 | | Princess et al.[11] | | Tamil Nadu | | 2017 | | 1 |
| 12 | | Shetty et al.[12] | | Karnataka | | 2008 | | 1 |
| 13 | | Bhat et al.[13] | | Karnataka | | 2023 | | 1 |
| 14 | | Garg et al.[14] | | Karnataka | | 2020 | | 7 |
| 15 | | Thorve et al.[15] | | Maharashtra | | 2023 | | 1 |
| 16 | | Dubey et al.[16] | | Uttar Pradesh | | 2023 | | 4 |
| 17 | | Pillai et al.[17] | | Kerala | | 2014 | | 2 |
| 18 | | Rodrigues et al.[18] | | Goa | | 2020 | | 1 |
| 19 | | Raji et al.[19] | | Tamil Nadu | | 2018 | | 1 |
| 20 | | Naha et al.[20] | | Karnataka | | 2014 | | 1 |
| 21 | | Viswaroop et al.[21] | | Tamil Nadu | | 2007 | | 2 |
| 22 | | Vemuri et al.[22] | | Pondicherry | | 2021 | | 1 |
| 23 | | Baikunje et al.[23] | | Karnataka | | 2021 | | 1 |
| 24 | | Saravu et al.[24] | | Karnataka | | 2015 | | 2 |
| 25 | | Deshmukh et al.[25] | | Maharashtra | | 2013 | | 1 |
| 26 | | Andraj et al.[26] | | Pondicherry | | 2012 | | 1 |
| 27 | | Sampath et al.[27] | | Madhya Pradesh | | 2023 | | 1 |
| 28 | | Singhal et al.[28] | | Delhi | | 2023 | | 1 |
| 29 | | Gupta et al.[29] | | Karnataka | | 2025 | | 9 |
| 30 | | Subramanian et al.[30] | | Tamil Nadu | | 2020 | | 3 |
| 31 | | Vidyalakshmi et al.[31] | | Karnataka | | 2008 | | 22 |
| 32 | | Patil et al.[32] | | Telangana | | 2024 | | 1 |
| 33 | | Kunthuparambil et al.[33] | | Kerala | | 2013 | | 1 |
| 34 | | Rudrabhatla et al.[34] | | Kerala | | 2022 | | 1 |
| 35 | | Ramamoorthi et al.[35] | | Karnataka | | 2013 | | 6 |
| 36 | | Pradhan et al.[36] | | Bihar | | 2019 | | 1 |
| 37 | | Prabhat et al.[37] | | Odisha | | 2019 | | 1 |
| 38 | | Mohan et al.[38] | | Tamil Nadu | | 2021 | | 2 |
| 39 | | James et al.[39] | | Tamil Nadu | | 2013 | | 1 |
| 40 | | Mansoor et al.[40] | | Kerala | | 2016 | | 1 |
| 41 | | Rajendran et al.[41] | | Tamil Nadu | | 2024 | | 3 |
| 42 | | Priyadharshini et al.[42] | | Tamil Nadu | | 2023 | | 1 |
| 43 | | Wadwekar et al.[43] | | Pondicherry | | 2018 | | 1 |
| 44 | | Jang et al.[44] | | Delhi | | 2024 | | 1 |
| 45 | | Mathew et al.[45] | | Karnataka | | 2020 | | 1 |
| 46 | | Teresa et al.[46] | | Karnataka | | 2013 | | 1 |
| 47 | | Perumal et al.[47] | | Tamil Nadu | | 2020 | | 26 |
| 48 | | Gautam et al.[48] | | Tamil Nadu | | 2020 | | 1 |
| 49 | | Gupta et al.[49] | | West Bengal | | 2021 | | 1 |
| 50 | | Tripathy et al.[50] | | Uttar Pradesh | | 2024 | | 1 |
| 51 | | Jakribettu et al.[51] | | Kerala | | 2014 | | 1 |
| 52 | | Naha et al.[52] | | Karnataka | | 2012 | | 1 |
| 53 | | Shobhana et al.[53] | | West Bengal | | 2022 | | 1 |
| 54 | | Tirlangi et al.[54] | | Karnataka | | 2025 | | 1 |
| 55 | | Veluthat et al.[55] | | Karnataka | | 2021 | | 1 |
| 56 | | Sebastian et al.[56] | | Karnataka | | 2024 | | 1 |
| 57 | | Patil et al.[57] | | Maharashtra | | 2021 | | 1 |
| 58 | | Agarwal et al.[58] | | Chhattisgarh | | 2023 | | 1 |
| 59 | | Krishmoorthy et al.[59] | | Tamil Nadu | | 2020 | | 1 |
| 60 | | D'silva et al.[60] | | Pondicherry | | 2018 | | 1 |
| 61 | | Patro et al.[61] | | Odisha | | 2019 | | 2 |
| 62 | | Jayaprakash et al.[62] | | Karnataka | | 2016 | | 1 |
| 63 | | Arockiaraj et al.[63] | | Tamil Nadu | | 2016 | | 4 |
| 64 | | Frincy et al.[64] | | Assam | | 2020 | | 2 |
| 65 | | Jamkhandi et al.[65] | | Tamil Nadu | | 2014 | | 2 |
| 66 | | Krishna et al. [66] | | Tamil Nadu | | 2024 | | 1 |
| 67 | | Subramaniam et al.[67] | | Pondicherry | | 2013 | | 1 |
| 68 | | Amin et al.[68] | | Telangana | | 2023 | | 8 |
| 69 | | Agrawal et al.[69] | | Maharashtra | | 2023 | | 1 |
| 70 | | Vithiya et al.[70] | | Tamil Nadu | | 2024 | | 22 |
| 71 | | Nair et al.[71] | | Kerala | | 2018 | | 1 |
| 72 | | Bhandary et al.[72] | | Karnataka | | 2018 | | 1 |
| 73 | | Devi et al.[73] | | Pondicherry | | 2024 | | 1 |
| 74 | | Vithiya et al.[74] | | Tamil Nadu | | 2024 | | 13 |
| 75 | | Jacob et al.[75] | | Kerala | | 2018 | | 1 |
| 76 | | Mukhopadhyay et al.[76] | | Karnataka | | 2010 | | 2 |
| 77 | | Mahapatra et al.[77] | | Odisha | | 2021 | | 1 |
| 78 | | Nivedhana et al.[78] | | Tamil Nadu | | 2016 | | 1 |
| 79 | | Shamim et al.[79] | | West Bengal | | 2024 | | 2 |
| 80 | | Shetty et al.[80] | | Karnataka | | 2022 | | 1 |
| 81 | | Nayak et al.[81] | | Goa | | 2018 | | 1 |
| 82 | | Kumar et al.[82] | | Rajasthan | | 2024 | | 6 |
| 83 | | Chowdhury et al.[83] | | Karnataka | | 2024 | | 1 |
| 84 | | Rajinikanth et al.[84] | | Tamil Nadu | | 2008 | | 1 |
| 85 | | Jabeen et al.[85] | | Karnataka | | 2021 | | 1 |
| 86 | | Kumari et al.[86] | | Tamil Nadu | | 2024 | | 3 |
| 87 | | Gupta et al.[87] | | Madhya Pradesh | | 2022 | | 2 |
| 88 | | Sovane et al.[88] | | Maharashtra | | 2024 | | 1 |
| 89 | | Indulekha et al.[89] | | Uttarakhand | | 2022 | | 1 |
| 90 | | Ekka et al.[90] | | Tamil Nadu | | 2017 | | 1 |
| 91 | | Mohapatra et al.[91] | | Chhattisgarh | | 2024 | | 1 |
| 92 | | Sivaselvi et al.[92] | | Pondicherry | | 2024 | | 1 |
| 93 | | Khatod et al.[93] | | Rajasthan | | 2024 | | 1 |
| 94 | | Uddandam et al.[94] | | Karnataka | | 2023 | | 1 |
| 95 | | Jain et al.[95] | | Odisha | | 2023 | | 1 |
| 96 | | Prasanna et al.[96] | | Maharashtra | | 2023 | | 3 |
| 97 | | Meenakumari et al.[97] | | Kerala | | 2023 | | 1 |
| 98 | | Naik et al.[98] | | Odisha | | 2023 | | 6 |
| 99 | | Kumar et al.[99] | | Rajasthan | | 2023 | | 1 |
| 100 | | Sharma et al.[100] | | Karnataka | | 2022 | | 3 |
| 101 | | Bhaskaran et al.[101] | | Kerala | | 2022 | | 2 |
| 102 | | Agrawal et al.[102] | | Maharashtra | | 2022 | | 1 |
| 103 | | Chandra et al.[103] | | Telangana | | 2022 | | 1 |
| 104 | | Shanmugam et al.[104] | | Tamil Nadu | | 2021 | | 1 |
| 105 | | Radhakrishnan et al.[105] | | Odisha | | 2021 | | 33 |
| 106 | | Sundaram et al.[106] | | Kerala | | 2021 | | 1 |
| 107 | | Muthina et al.[107] | | Andhra Pradesh | | 2021 | | 1 |
| 108 | | Iyer et al.[108] | | Telangana | | 2021 | | 1 |
| 109 | | Mishra et al.[109] | | Delhi | | 2021 | | 1 |
| 110 | | Chatterjee et al.[110] | | Karnataka | | 2021 | | 3 |
| 111 | | Gupta et al.[111] | | Karnataka | | 2021 | | 11 |
| 112 | | Kulkarni et al.[112] | | Tamil Nadu | | 2020 | | 2 |
| 113 | | Rao et al.[113] | | Karnataka | | 2020 | | 1 |
| 114 | | Rahman et al.[114] | | Odisha | | 2020 | | 2 |
| 115 | | Koshy et al.[115] | | Tamil Nadu | | 2019 | | 20 |
| 116 | | Annamalai et al.[116] | | Tamil Nadu | | 2019 | | 1 |
| 117 | | Gunasekaran et al.[117] | | Tamil Nadu | | 2018 | | 1 |
| 118 | | Kamath et al.[118] | | Karnataka | | 2018 | | 1 |
| 119 | | Garg et al.[119] | | Karnataka | | 2018 | | 1 |
| 120 | | Nair et al.[120] | | Kerala | | 2017 | | 1 |
| 121 | | Patil et al.[121] | | Karnataka | | 2017 | | 1 |
| 122 | | Antony et al.[122] | | Tamil Nadu | | 2017 | | 1 |
| 123 | | Kundangar et al.[123] | | Karnataka | | 2017 | | 1 |
| 124 | | Achappa et al.[124] | | Karnataka | | 2016 | | 1 |
| 125 | | Sathiavageesan et al.[125] | | Tamil Nadu | | 2016 | | 1 |
| 126 | | Madi et al.[126] | | Karnataka | | 2016 | | 1 |
| 127 | | Ray et al.[127] | | West Bengal | | 2016 | | 8 |
| 128 | | Vijaykumar et al.[128] | | Tamil Nadu | | 2016 | | 1 |
| 129 | | Amrutha et al.[129] | | Kerala | | 2016 | | 1 |
| 130 | | Mohanty et al.[130] | | Odisha | | 2016 | | 1 |
| 131 | | Vaid et al.[131] | | Karnataka | | 2015 | | 1 |
| 132 | | Garg et al.[132] | | Delhi | | 2015 | | 2 |
| 133 | | Padmaja et al.[133] | | Telangana | | 2015 | | 2 |
| 134 | | Shetty et al.[134] | | Karnataka | | 2015 | | 1 |
| 135 | | Neliyathodi et al.[135] | | Kerala | | 2015 | | 1 |
| 136 | | Sood et al.[136] | | Rajasthan | | 2014 | | 1 |
| 137 | | Totagi et al.[137] | | Tamil Nadu | | 2014 | | 2 |
| 138 | | Subramanyam et al.[138] | | Kerala | | 2014 | | 1 |
| 139 | | Krovvidi et al.[139] | | Andhra Pradesh | | 2013 | | 1 |
| 140 | | Esther et al.[140] | | Pondicherry | | 2013 | | 1 |
| 141 | | Boruah et al.[141] | | Assam | | 2013 | | 1 |
| 142 | | Bhat et al.[142] | | Karnataka | | 2012 | | 1 |
| 143 | | Prasad et al.[143] | | Pondicherry | | 2012 | | 1 |
| 144 | Rajadhyaksha et al.[144] | | Maharashtra | | 2012 | | 1 | |
| 145 | Behera et al.[145] | | Telangana | | 2012 | | 1 | |
| 146 | | Saravu et al.[146] | | Karnataka | | 2012 | | 1 |
| 147 | | Viswanathan et al.[147] | | Pondicherry | | 2012 | | 1 |
| 148 | | Shivbalan et al.[148] | | Tamil Nadu | | 2010 | | 1 |
| 149 | | Mukhopadhyay et al.[149] | | Karnataka | | 2010 | | 2 |
| 150 | | Kulkarni et al.[150] | | Karnataka | | 2010 | | 1 |
| 151 | | Shetty et al.[151] | | Karnataka | | 2010 | | 1 |
| 152 | | Kuruvilla et al.[152] | | Karnataka | | 2010 | | 1 |
| 153 | | Shenoy et al.[153] | | Karnataka | | 2009 | | 1 |
| 154 | | Valsalan et al.[154] | | Karnataka | | 2009 | | 2 |
| 155 | | Ray et al.[155] | | West Bengal | | 2009 | | 1 |
| 156 | | Noyal et al.[156] | | Pondicherry | | 2009 | | 1 |
| 157 | | Saravu et al.[157] | | Karnataka | | 2008 | | 6 |
| 158 | | Valsalan et al.[158] | | Karnataka | | 2008 | | 1 |
| 159 | | Kumar et al.[159] | | Tamil Nadu | | 2008 | | 6 |
| 160 | | Mukhopadhya et al.[160] | | Tamil Nadu | | 2007 | | 2 |
| 161 | | Dias et al.[161] | | Karnataka | | 2004 | | 1 |
| 162 | | Anuradha et al.[162] | | Telangana | | 2003 | | 1 |
| 163 | | Rao et al.[163] | | Karnataka | | 2002 | | 5 |
| 164 | | Rao et al.[164] | | Karnataka | | 1999 | | 1 |
| 165 | | Lath et al.[165] | | Tamil Nadu | | 1998 | | 1 |
| 166 | | Sanklecha et al.[166] | | Maharashtra | | 1997 | | 2 |
| 167 | | Cherian et al. [167] | | Tamil Nadu | | 1996 | | 1 |
| 168 | | Raghavan et al. [168] | | Maharashtra | | 1991 | | 1 |
| 169 | | Muthanikkatt et al.[169] | | Pondicherry | | 2025 | | 1 |
| 170 | | Gupta et al.[170] | | Karnataka | | 2024 | | 4 |
| 171 | | Devi et al.[171] | | Odisha | | 2025 | | 1 |
| 172 | | Gupta et al.[172] | | Karnataka | | 2024 | | 8 |
| 173 | | Waseem et al.[173] | | Tamil Nadu | | 2023 | | 1 |
| 174 | | Bhaskar et al.[174] | | Pondicherry | | 2023 | | 1 |
| 175 | | Harsha et al.[175] | | Karnataka | | 2023 | | 1 |
| 176 | | Jayakumar et al.[176] | | Pondicherry | | 2022 | | 1 |
| 177 | | Agrawal et al.[177] | | Maharashtra | | 2023 | | 1 |
| 178 | | Singhal et al.[178] | | Maharashtra | | 2022 | | 1 |
| 179 | | Bahuleyan et al.[179] | | Kerala | | 2022 | | 1 |
| 180 | | Singhai et al.[180] | | Madhya Pradesh | | 2022 | | 1 |
| 181 | | Deb et al.[181] | | Andaman and Nicobar | | 2021 | | 1 |
| 182 | | Mohanty et al.[182] | | Odisha | | 2022 | | 1 |
| 183 | | Halim et al.[183] | | Bihar | | 2021 | | 1 |
| 184 | | Nair et al.[184] | | Karnataka | | 2021 | | 1 |
| 185 | | Patel et al.[185] | | Gujarat | | 2021 | | 11 |
| 186 | | Maramattom et al.[186] | | Kerala | | 2021 | | 1 |
| 187 | | Savaj et al.[187] | | Gujarat | | 2020 | | 1 |
| 188 | | Kandhasamy et al.[188] | | Pondicherry | | 2020 | | 1 |
| 189 | | Parija et al.[189] | | Chhattisgarh | | 2020 | | 1 |
| 190 | | Subramony et al.[190] | | Tamil Nadu | | 2019 | | 1 |
| 191 | | Alexander et al.[191] | | Tamil Nadu | | 2018 | | 1 |
| 192 | | Saluja et al.[192] | | Tamil Nadu | | 2019 | | 1 |
| 193 | | Ninan et al.[193] | | Tamil Nadu | | 2018 | | 1 |
| 194 | | Gouse et al.[194] | | Tamil Nadu | | 2017 | | 18 |
| 195 | | Jagtap et al.[195] | | Telangana | | 2017 | | 9 |
| 196 | | Goel et al.[196] | | Delhi | | 2016 | | 1 |
| 197 | | Kumar et al.[197] | | Pondicherry | | 2017 | | 1 |
| 198 | | Karuna et al.[198] | | West Bengal | | 2015 | | 1 |
| 199 | | Tyagi et al.[199] | | Delhi | | 2014 | | 1 |
| 200 | | Pal et al.[200] | | West Bengal | | 2014 | | 1 |
| 201 | | Barman et al.[201] | | Delhi | | 2013 | | 1 |
| 202 | | Barman et al.[202] | | Delhi | | 2011 | | 1 |
| 203 | | Antony et al.[203] | | Karnataka | | 2010 | | 17 |
| 204 | | Pandey et al.[204] | | Karnataka | | 2010 | | 5 |
| 205 | | Dhodapkar et al.[205] | | Pondicherry | | 2008 | | 1 |
| 206 | | Thomas et al.[206] | | Telangana | | 2008 | | 1 |
| 207 | | Mathew et al.[207] | | Tamil Nadu | | 1999 | | 5 |
| 208 | | Mukhopadhyay et al.[208] | | Karnataka | | 2008 | | 25 |
| 209 | | Karthickeyan et al. [209] | | Karnataka | | 2014 | | 1 |

References

[1] Dash P, Prusty S, Pattnaik S, Mohapatra N. Solid Organ Abscess by Melioidosis: An Emerging Infection Among Diabetics in Odisha, India. CUREUS JOURNAL OF MEDICAL SCIENCE 2022;14. https://doi.org/10.7759/cureus.32975.

[2] Patil H, Gundavda M, Shetty V, Soman R, Rodriques C, Agashe V. Musculoskeletal melioidosis: An under-diagnosed entity in developing countries. JOURNAL OF ORTHOPAEDICS 2016;13:40–2. https://doi.org/10.1016/j.jor.2015.08.001.

[3] Easow J, Bhosale N, Pramodhini S, Priyadarshini R. Case Series of Melioidosis in a Tertiary Health Care Centre in Puducherry, India. JOURNAL OF PURE AND APPLIED MICROBIOLOGY 2024;18:185–92. https://doi.org/10.22207/JPAM.18.1.55.

[4] Pande A, Nambi P, Pandian S, Subramanian S, Ghosh S. Melioidosis mimicking tuberculous vertebral osteitis: Case report and review of literature. NEUROLOGY INDIA 2018;66:1100–5. https://doi.org/10.4103/0028-3886.236976.

[5] Mohanty S, Sarkar S, Mishra B. Melioidosis of the Head and Neck: A Case Series from Eastern India. INFECTIOUS DISEASE REPORTS 2020;12:36–45. https://doi.org/10.3390/idr12030011.

[6] Varma A, Mathew J, Gopalakrishnan C, Wilson A. Isolated Discitis in Melioidosis: An Unknown Presentation and a Microbiological Challenge. JOURNAL OF NEUROSCIENCES IN RURAL PRACTICE 2021;12:599–600. https://doi.org/10.1055/s-0041-1729477.

[7] Mamtora D, Davis A, Koppikar M, Cunha N, Bhalekar P. Burkholderia pseudomallei: Liver Abscess in a Diabetic Patient from Western India. JOURNAL OF CLINICAL AND DIAGNOSTIC RESEARCH 2018;12:DD06–7. https://doi.org/10.7860/JCDR/2018/34839.11275.

[8] Bojanapati K, Gohil R, Siddem C, Kumar J. Whitmore’s Disease: A Case Report of Melioidosis Triggering Hemophagocytic Lymphohistiocytosis. CUREUS JOURNAL OF MEDICAL SCIENCE 2024;16. https://doi.org/10.7759/cureus.65948.

[9] Singh A, Grover N, Gupta S, Bhatt P, Sahni A. Disseminated melioidosis: a case report. REVIEWS IN MEDICAL MICROBIOLOGY 2015;26:116–8. https://doi.org/10.1097/MRM.0000000000000034.

[10] Yadav V, Pawar A, Meena M, Khadanga S, Gupta A, Dandasena T, et al. Melioidosis as a Mystique Infection: A Study From Central India. CUREUS JOURNAL OF MEDICAL SCIENCE 2023;15. https://doi.org/10.7759/cureus.43439.

[11] Princess I, Ebenezer R, Ramakrishnan N, Daniel A, Nandini S, Thirunarayan M. Melioidosis: An Emerging Infection with Fatal Outcomes. INDIAN JOURNAL OF CRITICAL CARE MEDICINE 2017;21:397–400. https://doi.org/10.4103/ijccm.IJCCM_122_17.

[12] Shetty A, Hegde A, Shetty I, Gomes L. Cellulitis With Multiple Abscesses In Leg Due To Burkholderia Pseudomallei Infection-A Case Report. JOURNAL OF CLINICAL AND DIAGNOSTIC RESEARCH 2008;2:1196–9.

[13] Bhat V, Gosavi S, Krishnan G, Acharya R. A Case of Disseminated Melioidosis With Cerebritis. CUREUS JOURNAL OF MEDICAL SCIENCE 2023;15. https://doi.org/10.7759/cureus.40182.

[14] Garg R, Shaw T, Vandana K, Magazine R, Mukhopadhyay C. Melioidosis In Suspected Recurrent Tuberculosis: A disease in disguise. JOURNAL OF INFECTION IN DEVELOPING COUNTRIES 2020;14:312–6. https://doi.org/10.3855/jidc.12051.

[15] Thorve S, Mishra H, Waghmare S, Karpe S, Nair J. Melioidosis: Case report of rare pulmonary infection. PNEUMON 2023;36. https://doi.org/10.18332/pne/163487.

[16] Dubey D, Bano N, Dubey M, Sangwan P, Mitra S, Kulshrestha V, et al. A case series of melioidosis: An underdiagnosed infection*. IDCASES 2023;31. https://doi.org/10.1016/j.idcr.2023.e01685.

[17] Pillai M, Faizal B, Urs V. Emerging Drug Resistance in Melioidosis. ANNALS OF TROPICAL MEDICINE AND PUBLIC HEALTH 2014;7:263–5. https://doi.org/10.4103/1755-6783.155020.

[18] Rodrigues E, Dhupar V, Pinto M, Kumar P, Gurrala S. Jugular Lymphadenitis as a Precursor to Burkholderia Pseudomallei Sepsis. JOURNAL OF MAXILLOFACIAL & ORAL SURGERY 2020;19:370–3. https://doi.org/10.1007/s12663-020-01333-y.

[19] Raji V, Vasanthraj P, Ramachandran R, Sai V. Multi-system infection - tuberculosis or melioidosis? EGYPTIAN JOURNAL OF RADIOLOGY AND NUCLEAR MEDICINE 2018;49:689–92. https://doi.org/10.1016/j.ejrnm.2018.03.012.

[20] Naha K, Shastry B, Saravu K. Colonization or spontaneous resolution: Expanding the role for Burkholderia pseudomallei. ASIAN PACIFIC JOURNAL OF TROPICAL MEDICINE 2014;7:250–2. https://doi.org/10.1016/S1995-7645(14)60031-6.

[21] Viswaroop B, Balaji V, Mathai E, Kekre N. Melioidosis presenting as genitourinary infection in two men with diabetes. JOURNAL OF POSTGRADUATE MEDICINE 2007;53:108–10.

[22] Vemuri M, Malik A, Mohapatra M, Sistla S, Purath S, Raj S, et al. Pulmonary melioidosis complicating foreign body aspiration in a young adult. RESPIROLOGY CASE REPORTS 2021;9. https://doi.org/10.1002/rcr2.819.

[23] Baikunje N, Gangaramajalu S, Hosmane G. Melioidosis: A Fulminant Infection in a Patient with Uncontrolled Diabetes. JOURNAL OF HEALTH AND ALLIED SCIENCES NU 2021;11:50–3. https://doi.org/10.1055/s-0040-1721232.

[24] Saravu K, Kadavigere R, Shastry B, Pai R, Mukhopadhyay C. Neurologic melioidosis presented as encephalomyelitis and subdural collection in two male labourers in India. JOURNAL OF INFECTION IN DEVELOPING COUNTRIES 2015;9:1289–93. https://doi.org/10.3855/jidc.6586.

[25] Deshmukh M, Mundhada S. Chronic suppurative joint effusion due to burkholderia pseudomallei: A case report. INDIAN JOURNAL OF PATHOLOGY AND MICROBIOLOGY 2013;56:460–3. https://doi.org/10.4103/0377-4929.125373.

[26] Anandraj V, Priyadharshini A, Sunil S, Ambedkar R. Burkholderia pseudomallei infection in a healthy adult from a rural area of South India. INDIAN JOURNAL OF PATHOLOGY AND MICROBIOLOGY 2012;55:578–9. https://doi.org/10.4103/0377-4929.107829.

[27] Sampath A, Sukumar M, Tejaswini P, Gupta A, Gpt C, Khadanga S. Acute Pneumonia Like Illness and Sepsis in India: Is it Time to Suspect Pulmonary Melioidosis? CUREUS JOURNAL OF MEDICAL SCIENCE 2023;15. https://doi.org/10.7759/cureus.36122.

[28] Singhal A, Kompella K, Mahajan P, Kartik B. Melioidosis: A Rare Presentation of Oligoarthritis. INDIAN JOURNAL OF RHEUMATOLOGY 2023;18:322–5. https://doi.org/10.4103/injr.injr_95_22.

[29] Gupta N, Malla S, Boodman C, Kumar T, Varma M, Mukhopadhyay C. Abscesses due to Melioidosis: A case-based review. CURRENT RESEARCH IN MICROBIAL SCIENCES 2025;8. https://doi.org/10.1016/j.crmicr.2024.100321.

[30] Subramanian S, Jaganathan V, Hari D, Thangaraju D. Melioidosis-role of endobronchial ultrasound. RESPIROLOGY CASE REPORTS 2020;8. https://doi.org/10.1002/rcr2.634.

[31] Vidyalakshmi K, Chakrapani M, Shrikala B, Damodar S, Lipika S, Vishal S. Tuberculosis mimicked by melioidosis. INTERNATIONAL JOURNAL OF TUBERCULOSIS AND LUNG DISEASE 2008;12:1209–15.

[32] Patil C, Mangalagiri N, Ajmera P, Kumar K, Reddy H. A Case Report of Whitmore’s Disease: A True Masquerader. CUREUS JOURNAL OF MEDICAL SCIENCE 2024;16. https://doi.org/10.7759/cureus.52409.

[33] Kunnathuparambil S, Sathar S, Tank D, Sreesh S, Mukunda M, Narayanan P, et al. Splenic abscess due to chronic melioidosis in a patient previously misdiagnosed as tuberculosis. ANNALS OF GASTROENTEROLOGY 2013;26:77–9.

[34] Rudrabhatla P, Nair S, George J, Sekar S, Ponnambath D. Isolated Myelitis and Intramedullary Spinal Cord Abscess in Melioidosis-A Case Report. NEUROHOSPITALIST 2022;12:131–6. https://doi.org/10.1177/19418744211025386.

[35] Ramamoorthi K, Saravu K, Mukhyopadhyaya C, Barakur A. Melioidosis: an underdiagnosed disease in India (epidemiology, clinical features, and outcomes). ASIAN BIOMEDICINE 2013;7:249–56. https://doi.org/10.5372/1905-7415.0702.173.

[36] Pradhan S, Rout A, Sirka C, Das S, Sahu K. Melioidosis complicating lepromatous leprosy with type 2 lepra reaction: A rare case report from India. LEPROSY REVIEW 2019;90:450–5.

[37] Prabhat V, Gantaguru A, Behera S, Rana R, Das G. Spinal Epidural Abscess in Melioidosis: A Rare Case Report from Eastern India. CUREUS JOURNAL OF MEDICAL SCIENCE 2019;11. https://doi.org/10.7759/cureus.4187.

[38] Mohan V, Rajesh K, Srinivas S, Ravi R, Prabhakaran J, Srinivasan K, et al. Unusual Presentations of Abdominal Melioidosis. JOURNAL OF GLOBAL INFECTIOUS DISEASES 2021;13:52–5. https://doi.org/10.4103/jgid.jgid_129_20.

[39] James D, Madhuri V, Gahukamble A, Choudhrie L, Pancharatnam P. Burkholderia pseudomallei Osteomyelitis of the Metatarsal in an Infant. JOURNAL OF FOOT & ANKLE SURGERY 2013;52:370–3. https://doi.org/10.1053/j.jfas.2012.12.009.

[40] Abdulla M, Alungal J. Melioidosis with endocarditis and massive cerebral infarct. ITALIAN JOURNAL OF MEDICINE 2016;10:55–7. https://doi.org/10.4081/itjm.2015.589.

[41] Rajendran A, Ali N, Manikandan E, Balaji J, Ravichandran V. Unveiling the Varied Faces of Melioidosis: A Case Series with Diverse Pulmonary Presentations. INDIAN JOURNAL OF RESPIRATORY CARE 2024;13:132–5. https://doi.org/10.5005/jp-journals-11010-1102.

[42] Priyadharshini J, Banu M, Ramya P, Senita S. A Case Report on Septic Arthritis caused by Burkholderia pseudomallei. JOURNAL OF CLINICAL AND DIAGNOSTIC RESEARCH 2023;17:DD4–6. https://doi.org/10.7860/JCDR/2023/64073.18738.

[43] Wadwekar B, Ninan R, Bhat S, Devi S, Ramaya S, Kanungo R. Lid abscess: An unusual presentation of melioidosis. AUSTRALASIAN MEDICAL JOURNAL 2018;11:322–5. https://doi.org/10.21767/AMJ.2017.2809.

[44] Jang K, Sharma A. A Peculiar Case of Disseminated Melioidosis with Atypical Features Likely Linked to Bong/Water Pipe Use. JOURNAL OF GLOBAL INFECTIOUS DISEASES 2024;16:183–5. https://doi.org/10.4103/jgid.jgid_5_24.

[45] Mathew A, Avantsa R, Shrikrishna U. CT Findings in Melioidosis: A Case Report. JOURNAL OF CLINICAL AND DIAGNOSTIC RESEARCH 2020;14. https://doi.org/10.7860/JCDR/2020/43295.13483.

[46] Teresa Mathai K, Bhat K, Ashraf M, Sarawag M, Kumar K. Melioidosis with a Pericardial Effusion, which Relapsed as a Chest Wall Abscess: A Rare Presentation. JOURNAL OF CLINICAL AND DIAGNOSTIC RESEARCH 2013;7:746–8. https://doi.org/10.7860/JCDR/2013/5215.2902.

[47] Perumal R, Livingston A, Samuel S, Govindaraju S. Melioidosis of the Musculoskeletal System. MEDICAL PRINCIPLES AND PRACTICE 2020;29:121–7. https://doi.org/10.1159/000503021.

[48] Gautam K, Lijesh K, Jude J, Gupta R, Paul T. An uncommon cause of fever in a patient with hyperthyroidism. JOURNAL OF FAMILY MEDICINE AND PRIMARY CARE 2020;9:432–4. https://doi.org/10.4103/jfmpc.jfmpc_933_19.

[49] Gupta A, Halder R, Chakraborty M, Chakraborty P. Isolated splenic abscess due to melioidosis in type 1 diabetes mellitus: laboratory diagnosis of Burkholderia pseudomallei in resource-restricted setting. BMJ CASE REPORTS 2021;14. https://doi.org/10.1136/bcr-2020-238985.

[50] Tripathy S, Pal S, Gutte S. Primary cutaneous melioidosis - A rare case needing intensive care unit admission. SOUTHERN AFRICAN JOURNAL OF CRITICAL CARE 2024;40. https://doi.org/10.7196/SAJCC.2024.v40i2.1362.

[51] Jakribettu R, Boloor R, D’Souza R, Aithala S. Subcutaneous surprise. ANNALS OF MEDICAL AND HEALTH SCIENCES RESEARCH 2014;4:123–5. https://doi.org/10.4103/2141-9248.126620.

[52] Naha K, Dasari S, Kusugodlu R, Prabhu M. Cranial melioidosis with extradural extension after a fall in the bathroom. AUSTRALASIAN MEDICAL JOURNAL 2012;5:455–8. https://doi.org/10.4066/AMJ.2012.1374.

[53] Shobhana A, Datta A, Trivedi S. CNS Melioidosis: A Diagnostic Challenge. NEUROLOGY INDIA 2022;70:778–80. https://doi.org/10.4103/0028-3886.344620.

[54] Tirlangi P, Kiran S, Vandana K, Mukhopadhyay C, Kundu R, Pai A, et al. Adjunctive nivolumab in combination with antibiotic therapy for the management of refractory melioidosis in a patient with metastatic breast cancer and chemotherapy-induced pancytopenia. TRANSACTIONS OF THE ROYAL SOCIETY OF TROPICAL MEDICINE AND HYGIENE 2025. https://doi.org/10.1093/trstmh/trae142.

[55] Veluthat C, Venkatnarayan K, Padaki P, Krishnaswamy U. Case of melioidosis misdiagnosed as pulmonary tuberculosis. BMJ CASE REPORTS 2021;14. https://doi.org/10.1136/bcr-2021-242499.

[56] Sebastian A, Gupta N, Varma M, Mukhopadhyay C. Necklace sign in Melioidosis. JOURNAL OF TRAVEL MEDICINE 2024. https://doi.org/10.1093/jtm/taae147.

[57] Patil S, Gondhali G. Pulmonary Melioidosis Masquerading as Tuberculosis: A Case Report. ELECTRONIC JOURNAL OF GENERAL MEDICINE 2021;18. https://doi.org/10.29333/ejgm/11064.

[58] Agarwal S, Hafeez A, Rao S, Galhotra V, Das P. Oral Melioidosis in Odontogenic Keratocyst of Mandible. JOURNAL OF MAXILLOFACIAL & ORAL SURGERY 2023;22:543–9. https://doi.org/10.1007/s12663-022-01763-w.

[59] Krishnamoorthy A, Fernando E, Dorairaj P, Thimmaiah A, Kumar D. Challenges in management of disseminated melioidosis with endocarditis in a patient with chronic kidney disease. INDIAN JOURNAL OF NEPHROLOGY 2020;30:416–9. https://doi.org/10.4103/ijn.IJN_185_19.

[60] D’silva A, Jayannan J, Karnam A, Nair S, Vasanthi N. Bilateral Pneumothorax-Rare Manifestation of Melioidosis. JOURNAL OF CLINICAL AND DIAGNOSTIC RESEARCH 2018;12:OD5–6. https://doi.org/10.7860/JCDR/2018/37171.12057.

[61] Patro S, Panda S, Mishra D, Keerthi S. Burkholderia Infections in Diabetic Patients Emerging as a Challenge for Physicians: A Case Series. JOURNAL OF CLINICAL AND DIAGNOSTIC RESEARCH 2019;13:OD04–7. https://doi.org/10.7860/JCDR/2019/39889.12671.

[62] Jayaprakash B, Rao N, Patil N, Balaji O, Rau N, Varghese G. Melioidosis: A Rare Case of Hemoptysis with Pseudoaneurysm. RESEARCH JOURNAL OF PHARMACEUTICAL BIOLOGICAL AND CHEMICAL SCIENCES 2016;7:1977–81.

[63] Arockiaraj J, Karthik R, Jeyaraj V, Amritanand R, Krishnan V, David K, et al. Non-Caseating Granulomatous Infective Spondylitis: Melioidotic Spondylitis. ASIAN SPINE JOURNAL 2016;10:1065–71. https://doi.org/10.4184/asj.2016.10.6.1065.

[64] Frincy K, Biswajyoti B, Saikia S, Baruah M, Devi U. Burkholderia pseudomallei septic arthritis in Type-2 diabetes mellitus patients: Report of two cases. INDIAN JOURNAL OF MEDICAL MICROBIOLOGY 2020;38:222–5. https://doi.org/10.4103/ijmm.IJMM_20_74.

[65] Jamkhandi D, Alex R, George K. Melioidosis: A report of two cases. NATIONAL MEDICAL JOURNAL OF INDIA 2014;27:202–3.

[66] Krishna M, Lakshmanan S, Rajendran V, Senthil N, Ayub I. Pyrexia of Unknown Origin in a Young Male: Unmasking Melioidosis in a Tuberculosis-Endemic Setting. CUREUS J Med Sci. 2024 July 7;16(7). n.d.

[67] Subramaniam R, Karthikeyan V, Sistla S, Ali S, Sistla S, Ram D, et al. Melioidosis Presenting as Pseudomyxoma Peritonei: Yet Another Pretense of the Great Mimicker: An Unreported Entity. Surg Infect. 2013 Aug;14(4):415–7. n.d.

[68] Amin D, Chinta S, Kapoor A, Subbalaxmi M, Reddy D, Neelima A, et al. Imaging case series of melioidosis: the great masquerader. Egypt J Radiol Nucl Med. 2023 Feb 27;54(1). n.d.

[69] Agrawal U, Sirsat R, Shetty A, Rodrigues C, Sunavala A. A fatal misidentification: Burkholderia pseudomallei misidentified as Acinetobacter spp. INDIAN J Med Microbiol. 2023 Nov;46. n.d.

[70] Vithiya G, Preethi G, Sundaram P, Rajendran T. Musculoskeletal melioidosis-a retrospective review of 22 cases from a tertiary care centre in South Tamilnadu. INDIAN J Med Microbiol. 2024 Nov;52. n.d.

[71] Nair R, Joy S, Krishnamurtrthy D. Melioidosis: An Emerging Infectious Disease as a Cause of Parotid Abscess: A Case Report. J Clin Diagn Res. 2018 July;12(7):MD01-MD2. n.d.

[72] Bhandary S, Mani I, Aroor R, Bhat V. A Case Report of Meilodosis. NITTE Univ J Health Sci. 2018 Sept;8(3):34–6. n.d.

[73] Devi K, Kalaiarasi R, Sivaraman G, Lakra N, Sastry A. Melioidosis of the Parotid Gland: Lessons Learned from a Fatal Case. INDIAN J Otolaryngol HEAD NECK Surg. 2024 Nov 9; n.d.

[74] Vithiya G, Rajalakshmi P, Sundaram P, Rajendran T. Neuromelioidosis - A retrospective review of thirteen cases from a tertiary care centre from South India. INDIAN J Med Microbiol. 2024 Nov;52. n.d.

[75] Jacob N, Paul N, Pulikottil S, Sreekumary P. SEPTICAEMIC MELIOIDOSIS IN A DIABETIC PATIENT FROM A TERTIARY CARE CENTRE IN KERALA. J Evol Med Dent Sci-JEMDS. 2018 Oct 22;7(43):4695–6. n.d.

[76] Mukhopadhyay C, Chawla K, Vandana K, Krishna S, Saravu K. Pulmonary melioidosis in febrile neutropenia: the rare and deadly duet. Trop Doct. 2010 July;40(3):165–6. n.d.

[77] Mahapatra A, Nayak H, Mishra G, Kumar C, Panigrahi M, Behera B, et al. Melioidosis in pancreatic pseudocyst: Atypical infection at atypical site. PANCREATOLOGY. 2021 Aug;21(5):1014–6. n.d.

[78] Nivedhana S, Rajendran S. Neonatal Melioidosis with Pneumatoceles. INDIAN Pediatr. 2016 Apr;53(4):352–352. n.d.

[79] Shamim P, Ahmed A, Mullick J. Intraoperative Cardiovascular Emergency in Spinal Melioidosis Presenting as a Case of Spinal Epidural Abscess: A Rare Case Study of 2 Patients. INDIAN J Microbiol. 2024 Sept;64(3):1099–109. n.d.

[80] Shetty N, Karikal A, Radhakrishnan S. Melioidosis: an unusual diagnosis for deep temporal and masticatory space infection following trauma. BMJ CASE Rep. 2022 June;15(6). n.d.

[81] Nayak C, Furtado N, Gomes E, Kulkarni S. RELAPSE OF THE BUG AT A DIFFERENT SITE- A CASE OF MULTIDRUG RESISTANT MELIOIDOSIS. J Evol Med Dent Sci-JEMDS. 2018 Apr 23;7(17):2163–4. n.d.

[82] Kumar S, Bhambhu V, Gugale S, Goyal R, Kalia A. Emerging Burkholderia Musculoskeletal Infections With Delayed Diagnosis in Non-endemic Regions Affect Patient Morbidity: A Case Series of 10 Patients With a Review of the Literature. CUREUS J Med Sci. 2024 Jan 28;16(1). n.d.

[83] Chowdhury S, Gupta N, Varma M, Eshwara V, Mukhopadhyay C. Melioidosis: a stain in time saves a life. QJM- Int J Med. 2024 July 8;117(10):747–8. n.d.

[84] Rajinikanth J, Balaji V, Gaikwad P, Muthusami J. Melioidosis of the parotid: The tip of the iceberg. Otolaryngol-HEAD NECK Surg. 2008 Nov;139(5):731–2. n.d.

[85] Jabeen S, Saini J, Mishra T, Mailankody P, Lingaraju T, Chandrashekar N. Neuromelioidosis Presenting as a Stroke-like Syndrome. Neurol-Clin Pract. 2021 Aug;11(4):E589–91. n.d.

[86] Kumari S, Banu M, Ramanatha K, Barani R, Sridharan K. Are Burkholderia Emerging Pathogens in patients with underlying morbidity: A case series. HELIYON. 2024 June 30;10(12). n.d.

[87] Gupta A, Siddiqui F, Purwar S, Joshi R, Mukhopadhyay C. Is it always COVID-19 in acute febrile illness in the tropics during the pandemic? PLoS Negl Trop Dis. 2022 Nov;16(11). n.d.

[88] Sonavane S, Jamale T, Bose S, Basu S. Febrile Immunocompromised Renal Transplant Recipient with Allograft Dysfunction: Detection of an Undiagnosed Prostate Abscess by [18F]FDG-PET/CT along with Treatment Response Monitoring. WORLD J Nucl Med. 2024 Sept;23(03):207–11. n.d.

[89] Indulekha H, Chandrakar S, Patnaik I, Patel N. Rare case of burkholderia liver abscess complicated by hepatobronchial fistula. BMJ CASE Rep. 2022 Jan;15(1). n.d.

[90] Ekka A, Mohideen M, Kesavan S. Neuromelioidosis Masquerading as Acute Demyelinating Encephalomyelitis. INDIAN Pediatr. 2017 Dec;54(12):1054–5. n.d.

[91] Mohapatra A, Agarwala P, Sirigiri HP, Das P. Disseminated melioidosis—challenge to routine antibiotic therapy: a case report. J Med Case Rep [Internet]. 2024;18(1). Available from: https://www.embase.com/search/results?subaction=viewrecord&id=L2031771700&from=export U2 - L2031771700 n.d.

[92] Sivaselvi C, Rajaram M, Warrier LS, Upadhya P. Burkholderia pseudomallei - an unusual cause of septic embolism. Chest Rep [Internet]. 2024;12(1). Available from: https://www.embase.com/search/results?subaction=viewrecord&id=L2029293876&from=export U2 - L2029293876 n.d.

[93] Khatod Y, Ramankutty N, Kumar D, Tak V, Gupta N, Rajagopal R, et al. Shoulder shake-up: Unveiling the unusual case of acute melioidosis. IDCASES. 2024;37. n.d.

[94] Uddandam A, Nandakrishna B, Acharya V, Sukumar CA. The Bug Story: Melioidosis with Candidaemia. J Indian Acad Clin Med. 2023;24(3):224–7. n.d.

[95] Jain M, Ratna HVK, Mohanty S, Padhi S, Tripathy S. Coinfection of Melioidosis and Tuberculosis Causing Infective Lumbar Spondylodiscitis: A Rare Case Report. JBJS Case Connect [Internet]. 2023;13(2). Available from: https://www.embase.com/search/results?subaction=viewrecord&id=L2024624941&from=export U2 - L2024624941 n.d.

[96] Prasanna S, Mahajan M, Mahajan N. CASE SERIES OF BURKHOLDERIA PSEUDOMALLEI CAUSING MELIOIDOSIS (PULMONARY, CEREBRAL, AND SPLENIC ABSCESS). Asian J Pharm Clin Res. 2023;16(5):4–6. n.d.

[97] Meenakumari PB, Rekha R, Neena PS, Suresh R. Burkholderia Pseudomallei Infection in a Diabetic Patient Presenting as Multiple Splenic Abscesses — A Case Report. J Indian Med Assoc. 2023;121(4):63–5. n.d.

[98] Naik S, Bhoi S, Jha M, Kumar M. Craniospinal MRI Findings in Neuromelioidosis. Neurol INDIA. 2023 Jan;71(1):113–8. n.d.

[99] Kumar P, Birda CL, Chand YS, Yadav T, Agarwal A. Melioidosis presenting as splenic abscess: Case report and review of literature. Indian J Gastroenterol. 2023;42:S118. n.d.

[100] Sharma A, Mahajan Z, Madhyastha S, Mehta V. Critical approach to atypical spectrum of melioidosis: a case-series based literature review. BMJ CASE Rep. 2022 June;15(6). n.d.

[101] Bhaskaran P, Prasad V, Gopinathan A, Shaw T, Sivadas S, Jayakumar C, et al. Burkholderia pseudomallei in Environment of Adolescent Siblings with Melioidosis, Kerala, India, 2019. Emerg Infect Dis. 2022 June;28(6):1246–9. n.d.

[102] Agrawal U, Samant R, Kothari J, Sunavala A. Melioidosis: Missed opportunities and opportunistic pathogens. Med J Armed Forces India. 2022;78(2):239–42. n.d.

[103] Chandra K, Rajesh BJ. Concomitant extradural, subdural, and intraparenchymal abscesses of the brain in a patient with cerebral melioidosis - A case report. Surg Neurol Intl [Internet]. 2022;13. Available from: https://www.embase.com/search/results?subaction=viewrecord&id=L2022340990&from=export U2 - L2022340990 n.d.

[104] Shanmugam S, Ramesh R, Kumar R, Radhan P, Kumar B. Preferential corticospinal tract involvement in a case of neuromelioidosis. Ann Indian Acad Neurol. 2021;24(6):927–8. n.d.

[105] Radhakrishnan A, Behera B, Mishra B, Mohapatra P, Kumar R, Singh A. Clinico-microbiological description and evaluation of rapid lateral flow immunoassay and PCR for detection of Burkholderia pseudomallei from patients hospitalized with sepsis and pneumonia: A twenty-one months study from Odisha, India. ACTA Trop. 2021 Sept;221. n.d.

[106] Sundaram P, Padma S, Chauhan R. 99mTc-MDP whole body bone scan in a case of acute disseminated melioidosis. Iran J Nucl Med. 2021 SUM;29(2):104–7. n.d.

[107] Muthina R, Koppara N, Manuel M, Bommu A, Anapalli S, Boju S, et al. Cerebral abscess and calvarial osteomyelitis caused by Burkholderia pseudomallei in a renal transplant recipient. Transpl Infect Dis. 2021 Apr;23(2). n.d.

[108] Iyer R, Jangam R, Nara B, Kondeti K. Multiple hepatic and splenic abscesses due to Burkholderia pseudomallei. INDIAN J Med Microbiol. 2021 Apr;39(2):249–51. n.d.

[109] Mishra B, Vishnu V, Bhatia R, Garg A, Doddamani R, Singh P, et al. Case Report: Isolated Central Nervous System Melioidosis from a Non-Endemic Area. Am J Trop Med Hyg. 2021 Apr;104(4):1247–51. n.d.

[110] Chatterjee A, Saravu K, Mukhopadhyay C, Chandran V. Neurological Melioidosis Presenting as Rhombencephalitis, Optic Neuritis, and Scalp Abscess with Meningitis: A Case Series from Southern India. Neurol INDIA. 2021 Mar;69(2):480–2. n.d.

[111] Gupta N, Bhat SN, Reddysetti S, Kadavigere R, Godkhindi VM, Mukhopadhyay C, et al. Osteoarticular melioidosis: A retrospective cohort study of a neglected disease. Infez Med. 2021;29(4):574–82. n.d.

[112] Kulkarni P, Shelley S, Elangoven I, Jaykanth A, Ejaz A, Rao N. 18-Fluorine-fluorodeoxyglucose positron emission Tomography-Computed tomography in the evaluation of the great masquerader melioidosis: A case series. INDIAN J Nucl Med. 2020 July;35(3):222–5. n.d.

[113] Rao S, Kamath N. Melioidosis Presenting with Membranous Tonsillitis and Erythema Nodosum. INDIAN Pediatr. 2020 May;57(5):482–3. n.d.

[114] Mujeeb Rahman KK, Bhuniya S, Behera B, Mohapatra PR, Kumar R, Radhakrishnan A. Utility of endobronchial ultrasound guided trans-bronchial needle aspiration in diagnosis of melioidosis - case series and review of literature. Monaldi Arch Chest Dis. 2020;90(3):420–4. n.d.

[115] Koshy M, Sadanshiv P, Sathyendra S. Genitourinary melioidosis: a descriptive study. Trop Doct. 2019 Apr;49(2):104–7. n.d.

[116] Annamalai A, Padmini K. Melioidosis. INDIAN J Med Res. 2019 Apr;149(4):561–2. n.d.

[117] Gunasekaran K, Amladi A, Mathew S, Miraclin T, Iyyadurai R. A case of septicaemic melioidosis: Utility of therapeutic drug monitoring and high-dose meropenem in successful management. INDIAN J Med Microbiol. 2018 Oct;36(4):597–9. n.d.

[118] Kamath SJ, Kolavala B, Saini V. Varied orbital inflammations in diabetics. J Clin Diagn Res. 2018;12(8):ND01–2. n.d.

[119] Garg R, Shaw T, Bhat SN, Mukhopadhyay C. Melioidosis: The great mimicker presenting as spondylodiscitis. BMJ Case Rep [Internet]. 2018;2018. Available from: https://www.embase.com/search/results?subaction=viewrecord&id=L620602555&from=export U2 - L620602555 n.d.

[120] Nair AV, Menon V, Kumaran CK, Khan PS, Kochukunju BV, Kurian N. Arthroscopic management of a rare case of melioidotic septic arthritis of the ankle in a new endemic area on the southwest coast of India: A case report. JBJS Case Connect [Internet]. 2017;7(3). Available from: https://www.embase.com/search/results?subaction=viewrecord&id=L617304878&from=export U2 - L617304878 n.d.

[121] Patil NA, Balaji O, Rao KN, Hande HM, Ahmed T, Singhal S. A rare cause of septic arthritis with pleural effusion: Burkholderia pseudomallei. Asian J Pharm Clin Res. 2017;10(1):8–9. n.d.

[122] Antony T, Moorthy S, Narayanaswamy A, Arthur P. Melioidosis presenting as septicaemia and facial nerve palsy. BMJ Case Rep [Internet]. 2017;2017. Available from: https://www.embase.com/search/results?subaction=viewrecord&id=L618574085&from=export U2 - L618574085 n.d.

[123] Kundangar RS, Bhat SN, Mohanty SP. Melioidosis mimicking tubercular cold abscess. BMJ Case Rep [Internet]. 2017;2017. Available from: https://www.embase.com/search/results?subaction=viewrecord&id=L619344364&from=export U2 - L619344364 n.d.

[124] Achappa B, Madi D, Vidyalakshmi K. Cutaneous Melioidosis. J Clin Diagn Res. 2016 Sept;10(9):WD1–2. n.d.

[125] Sathiavageesan S. Septicemic melioidosis in a transplant recipient causing graft dysfunction. Indian J Nephrol. 2016;26(5):379–82. n.d.

[126] Madi D, Rai S, Vidyalakshmi K, Chowta K. Neurological melioidosis presenting as intracranial abscess. INDIAN J Pathol Microbiol. 2016 July;59(3):417–9. n.d.

[127] Ray U, Dutta S, Ramasubban S, Sen D, Tiwary IK. Melioidosis: Series of Eight Cases. J Assoc Physicians India. 2016;64(5):42–6. n.d.

[128] Vijaykumar GS, Thilakavathy P, Jeremiah SS, Vithiya G. Osteomyelitis of humerus and intramuscular abscess due to melioidosis. Kathmandu Univ Med J. 2016;14(54):184–5. n.d.

[129] Amrutha M, Rajagopal T. Cutaneous melioidosis. QJM- Int J Med. 2016 Feb;109(2):129–129. n.d.

[130] Mohanty S, Pradhan G, Panigrahi MK, Mohapatra PR, Mishra B. A case of systemic melioidosis: unravelling the etiology of chronic unexplained fever with multiple presentations. Pneumonol Alergol Pol. 2016;84(2):121–5. n.d.

[131] Vaid T, Rao K, Hande HM. An intriguing case of locked jaw secondary to melioidosis. BMJ Case Rep [Internet]. 2015;2015. Available from: https://www.embase.com/search/results?subaction=viewrecord&id=L607628125&from=export U2 - L607628125 n.d.

[132] Garg G, Chawla N, Chawla K, Khosla P, Jain S. Atypical Presentations of Melioidosis in North India: Report of Two Cases. J Assoc Physicians India. 2015;63(6):82–3. n.d.

[133] Padmaja K, Lakshmi V, Sudhaharan S, Malladi SVS, Gopal P, Ravinuthala KV. Unusual presentation of melioidosis in a case of pseudoaneurysm of descending thoracic aorta: Review of two case reports. Res Cardiovasc Med [Internet]. 2015;4(2). Available from: https://www.embase.com/search/results?subaction=viewrecord&id=L604836460&from=export U2 - L604836460 n.d.

[134] Shetty HS, Mallela AR, Shastry BA, Acharya V. Parietal bone osteomyelitis in melioidosis. BMJ Case Rep [Internet]. 2015;2015. Available from: https://www.embase.com/search/results?subaction=viewrecord&id=L604164690&from=export U2 - L604164690 n.d.

[135] Neliyathodi S, Thazhathethil A, Pallivalappil L, Balakrishnan D. Pleuropulmonary melioidosis with osteomyelitis rib. LUNG INDIA. 2015 Jan;32(1):67–9. n.d.

[136] Sood S, Khedar R, Joad S, Gupta R. Septicaemic Melioidosis: Case Report from a Non-Endemic Area. J Clin Diagn Res. 2014 Dec;8(12):DD1–2. n.d.

[137] Totagi AB, Paramasivan P. Melioidosis: an unusual cause of isolated liver abscess. Trop Gastroenterol. 2014;35(4):261–3. n.d.

[138] Subramanyam P, Palaniswamy S. Multifocal Bone and Visceral Melioidosis in a Cirrhotic Patient Identified by 99mTc MDP Bone Scan. Am J Trop Med Hyg. 2014 Feb;90(2):191–191. n.d.

[139] Krovvidi R, Mridula R, Jabeen S, Meena A. Guillain Barre syndrome as a manifestation of neurological melioidosis. Ann INDIAN Acad Neurol. 2013 Oct;16(4):681–3. n.d.

[140] Esther P, Sudhagar M, Anandhalakshmi S, Shanthi M. A case report of melioidosis in a diabetic patient in a union territory. Australas Med J. 2013;6(8):401–5. n.d.

[141] Boruah DK, Prakash A, Bora R, Buragohain L. Acute pulmonary melioidosis in a child: A case report and review of literature. Indian J Radiol Imaging. 2013;23(4):310–2. n.d.

[142] Bhat S, Alva J, Ashraf M, Hiremath S, Duble S. Pericardial effusion - melioidosis or tuberculosis. J Med. 2012;13(2):230–2. n.d.

[143] Vishnu Prasad NR, Balasubramaniam G, Karthikeyan VS, Ramesh CK, Srinivasan K. Melioidosis of chest wall masquerading as a tubercular cold abscess. J Surg Tech Case Rep. 2012;4(2):115–7. n.d.

[144] Rajadhyaksha A, Sonawale A, Khare S, Kalal C, Jankar R. Disseminated melioidosis presenting as septic arthritis. J AssocPhys India. 2012;60(6):44–5. n.d.

[145] Behera B, Babu T, Kamalesh A, Reddy G. Ceftazidime resistance in Burkholderia pseudomallei: First report from India. ASIAN Pac J Trop Med. 2012 Apr;5(4):329–30. n.d.

[146] Saravu K, Mukhopadhyay C, Eshwara VK, Shastry BA, Ramamoorthy K, Krishna S, et al. Melioidosis presenting with mediastinal lymphadenopathy masquerading as malignancy: A case report. J Med Case Rep [Internet]. 2012;6. Available from: https://www.embase.com/search/results?subaction=viewrecord&id=L51829799&from=export U2 - L51829799 n.d.

[147] Viswanathan S, Remalayam B, Muthu V, Kumar S. Diabetes with multiple abscesses disseminated in time and place. Asian Pac J Trop Biomed. 2012;2(2):S1209–11. n.d.

[148] Shivbalan S, Reddy N, Tiru V, Thomas K. Systemic Melioidosis Presenting as Suppurative Parotitis. INDIAN Pediatr. 2010 Sept;47(9):799–801. n.d.

[149] Mukhopadhyay C, Vandana KE, Krishna S, Saravu K, Shastri BA. Aquatic to pulmonary: Severe melioidosis following near-drowning from Southern India. Internet J Pulm Med [Internet]. 2010;11(2). Available from: https://www.embase.com/search/results?subaction=viewrecord&id=L358693144&from=export U2 - L358693144 n.d.

[150] Kulkarni R, Jain P, Ajantha G, Shetty J, Chunchanur S, Shubhada C. Fatal Burkholderia pseudomallei septicaemia in a patient with diabetes. INDIAN J Med Res. 2010 Apr;131(4):584–5. n.d.

[151] Shetty A, Boloor R, Sharma V, Bhat G. Melioidosis and pulmonary tuberculosis co-infection in a diabetic. Ann Thorac Med. 2010 Apr;5(2):113–5. n.d.

[152] Kuruvilla T, Dias M, Udayan U, Furtado Z. Melioidotic pericardial effusion. Indian J Med Sci. 2010;64(2):94–8. n.d.

[153] Shenoy V, Kamath M, Hegde M, D’Souza T, Mammen S. Melioidosis and tuberculosis: dual pathogens in a neck abscess. J Laryngol Otol. 2009 Nov;123(11):1285–7. n.d.

[154] Valsalan R, Shubha S, Mukhopadhyay C, Saravu K, Maneesh M, Shastry BA, et al. False-positive widal in melioidosis. Indian J Med Sci. 2009;63(10):464–7. n.d.

[155] Ray U, Sen D, Kar S. Septicaemic melioidosis. J AssocPhys India. 2009;57(8):598–9. n.d.

[156] Noyal M, Harish B, Bhat V, Parija S. Neonatal melioidosis: A case report from India. INDIAN J Med Microbiol. 2009 July;27(3):260–3. n.d.

[157] Saravu K, Vishwanath S, Kumar RS, Barkur AS, Varghese GK, Mukhyopadhyay C, et al. Melioidosis - a case series from south India. Trans R Soc Trop Med Hyg. 2008;102:S18–20. n.d.

[158] Valsalan R, Seshadri S, Pandit VR. Melioidosis masquerading as enteric fever. Trans R Soc Trop Med Hyg. 2008;102:S117–8. n.d.

[159] Kumar G, Raj P, Chacko G, Lalitha M, Chacko A, Rajshekhar V. Cranial melioidosis presenting as a mass lesion or osteomyelitis. J Neurosurg. 2008 Feb;108(2):243–7. n.d.

[160] Mukhopadhya A, Balaji V, Jesudason M, Amte A, Jeyamani R, Kurian G. Isolated liver abscesses in melioidosis. Indian J Med Microbiol. 2007;25(2):150–1. n.d.

[161] Dias M, Antony B, Aithala S, Hanumanthappa B, Pinto H, Rekha B. Burkholderia pseudomallei septicaemia - A case report. Indian J Med Microbiol. 2004;22(4):266–8. n.d.

[162] Anuradha K, Meena AK, Lakshmi V. Isolation of Burkholderia pseudomallei from a case of septicaemia - A case report. Indian J Med Microbiol. 2003;21(2):129–32. n.d.

[163] Rao P, Dhawan R, Shivananda P. Burkholderia pseudomallei infections. Trop Doct. 2002 July;32(3):174–5. n.d.

[164] Rao PS, Shivananda PG. Burkholderia pseudomallei--abscess in an unusual site. Indian J Pathol Microbiol. 1999;42(4):493–4. n.d.

[165] Lath R, Rajshekhar V, George V. Brain abscess as the presenting feature of melioidosis. Br J Neurosurg. 1998 Apr;12(2):170–2. n.d.

[166] Sanklecha MU, Raghavan K, Mehta MN. Melioidosis--rare or overlooked? Indian J Pediatr. 1997;64(2):253–5. n.d.

[167] Cherian T, John TJ, Ramakrishna B, Lalitha MK, Raghupathy P. Disseminated melioidosis. Indian Pediatr. 1996;33(5):403–6. n.d.

[168] Raghavan KR, Shenoi RP, Zaer F, Aiyer R, Ramamoorthy P, Mehta MN. Melioidosis in India. Indian Pediatr. 1991;28(2):184–8. n.d.

[169] Muthanikkatt AM, Nathan B, Uthayakumar A, Devendiran A, Muthu S. Melioidosis - An under-recognized dreaded disease in Southeast Asia. Turk J Emerg Med. 2025;25(1):63–6. n.d.

[170] Gupta N, Malla S, Kumar T, Singh S, Varma M, Mukhopadhyay C. Tunnel sign in patients with melioidosis: a case series from South India. Trans R Soc Trop Med Hyg. 2024 Nov 18; n.d.

[171] Devi S, Dash A, Dey A, Patra S, Sahoo B, Mahapatra A, et al. Hemophagocytic lymphohistiocytosis complicating septicemic melioidosis: A case report. J Infect Chemother. 2025 Jan;31(1). n.d.

[172] Gupta N, Malla S, Tirlangi P, Magazine R, Uk C, Ravindra P, et al. Reversed halo sign: Do not forget Melioidosis. J TRAVEL Med. 2024 Mar 22;31(5). n.d.

[173] Al Waseem SMH, Antony T, Suresh S, Gopalan S. Haemophagocytic lymphohistiocytosis due to Burkholderia pseudomallei in a primigravida. Access Microbiol [Internet]. 2023;5(9). Available from: https://pubmed.ncbi.nlm.nih.gov/37841105/ n.d.

[174] Bhaskar M M, Rajamanikam S, Raj S, Sistla S, Nichanahalli KS. A rare and fatal case of tubo-ovarian abscess due to Burkholderia pseudomallei presenting as puerperal sepsis. Access Microbiol [Internet]. 2023;5(9). Available from: https://pubmed.ncbi.nlm.nih.gov/37841101/ n.d.

[175] Harsha M, Kumar T, Varma M, Mukhopadhyay C. Dealing with the honey trap: a case of hepatic melioidosis. QJM- Int J Med. 2023 Nov 24;116(11):940–1. n.d.

[176] Jayakumar B, P AJ. A Rare Case of Burkholderia Osteomyelitis Affecting the Hip Joint in an Adult. J Orthop Case Rep. 2022;12(12):77–82. n.d.

[177] Agrawal U, Sunavala A, Vaidya V, Roy R, Basu S, Drego L, et al. Ceftazidime-resistant Burkholderia pseudomallei masquerading as isolated atypical neuromelioidosis. INDIAN J Med Microbiol. 2023 Jan;41:111–3. n.d.

[178] Singhal T, Sonawane R, Kulkarni B, Raut A, Soman R. The mystery behind a 1000 day fever in a young male. INDIAN J Med Microbiol. 2022 Oct;40(4):596–8. n.d.

[179] Bahuleyan B, Adarsh M, Akarsh J, M L AK, Rohitha CS, Elenjickal GX, et al. Cerebral venous sinus thrombosis as a complication of cranial melioidosis - a rare case report. Access Microbiol. 2022;4(5):acmi000357. n.d.

[180] Singhai A, Mallik M, Ingle V, Mishra VN. A Rare Case of Acute Disseminated Melioidosis Following Lower Segment Caesarean Section. J Obstet Gynaecol India. 2022;72(3):258–61. n.d.

[181] Deb S, Singh M, Choudhary J, Jain VK, Kumar S. A Rare Case of a Knee Septic Arthritis by Burkholderia pseudomallei : A Case Report from a Tertiary Care Hospital of Andaman and Nicobar Island. J Orthop Case Rep. 2021;11(9):20–3. n.d.

[182] Mohanty S, Devi S, Saha S. Post-COVID-19 vaccination shoulder abscess and pleuroparenchymal pulmonary lesion due to Burkholderia pseudomallei. IDCASES. 2022;27. n.d.

[183] Halim I, Kokkayil P, Kirti R, Priyadarshi RN, Sarfraz A, Pati BK, et al. Melioidosis in Bihar, India: unearthing the first of many? Access Microbiol. 2021;3(9):000260. n.d.

[184] Nair S, Varsha N, Sunil H. Melioidosis Presenting as Septic Arthritis: The Role of F-18 Fludeoxyglucose Positron Emission Tomography/Computed Tomography in Diagnosis and Management. INDIAN J Nucl Med. 2021 Jan;36(1):59–61. n.d.

[185] Patel A, Dharsandiya M, Savaj P, Munim FC, Shah K, Patel K. Footprints of human melioidosis in the north western Indian states, expanding endemicity in India. Indian J Med Microbiol. 2021;39(3):373–5. n.d.

[186] Maramattom B, Rathish B. Case Report: Ascending Myelo-Encephalitis after a Penetrating Injury to the Foot: An Atypical Case of Neuromelioidosis. Am J Trop Med Hyg. 2021 Apr;104(4):1260–4. n.d.

[187] Savaj P, Shaw T, Munim F, Mukhopadhyay C. Melioidosis in Gujarat: a neglected tropical disease. Trop Doct. 2020 Oct;50(4):373–5. n.d.

[188] Chinnakkulam Kandhasamy S, Elamurugan TP, Naik D, Rohith G, Nelamangala Ramakrishnaiah VP. Systemic Melioidosis With Ruptured Splenic Abscess. Cureus. 2020;12(5):e7956. n.d.

[189] Parija D, Kar BK, Das P, Mishra JK, Agrawal AC, Yadav SK. Septic arthritis of knee due to Burkholderia pseudomallei: a case report. Trop Doct. 2020;50(3):254–7. n.d.

[190] Subramony H, Gunasekaran S, Pandit V. Disseminated melioidosis with native valve endocarditis: a case report. Eur HEART J-CASE Rep. 2019 June;3(2). n.d.

[191] Alexander V, Koshy M, Shenoy R, Sudarsanam TD. Chronic multifocal osteomyelitis: A rare presentation of melioidosis. J Fam Med Prim Care. 2018;7(5):1133–5. n.d.

[192] Saluja SS, Kumar MM, Gopal S. A Rare Case of Melioidosis Causing Multifocal Osteomyelitis in an Uncontrolled Diabetic Host. J Orthop Case Rep. 2019;9(5):95–101. n.d.

[193] Ninan F, Mishra AK, John AO, Iyadurai R. Splenic granuloma: Melioidosis or Tuberculosis? J Fam Med Prim Care. 2018;7(1):271–3. n.d.

[194] Gouse M, Jayasankar V, Patole S, Veeraraghavan B, Nithyananth M. Clinical Outcomes in Musculoskeletal Involvement of Burkholderia Pseudomallei Infection. Clin Orthop Surg. 2017 Sept;9(3):386–91. n.d.

[195] Jagtap N, Shah H, Kancharla A, Tandan M, Pal P, Lakhtakia S, et al. Gastrointestinal manifestations of melioidosis: A single center experience. INDIAN J Gastroenterol. 2017 Mar;36(2):141–4. n.d.

[196] Goel A, Bansal R, Sharma S, Singhal S, Kumar A. Chronic melioidosis presenting with multiple abscesses. Oxf Med Case Rep. 2016;2016(6):113–6. n.d.

[197] Kumar M, Krishnamurthy S, Venkateswaran V, Mahadevan S, Lalitha M, Sistla S, et al. Brainstem micro-abscesses caused by Burkholderia pseudomallei in a 10-month-old infant: a case report. Paediatr Int CHILD Health. 2017;37(3):230–2. n.d.

[198] Karuna T, Khadanga S, Dugar D, Sau B, Bhoi P. Melioidosis as a cause of acute abdomen in immuno-competent male from eastern India. J Lab Physicians. 2015;7(1):58–60. n.d.

[199] Tyagi P, Shah V, Sharma P, Bansal N, Singla V, Kumar A, et al. Melioidosis Presenting as Fever and Jaundice: A Rare Presentation. J Clin Exp Hepatol. 2014 June;4(2):172–4. n.d.

[200] Pal P, Ray S, Moulick A, Dey S, Jana A, Banerjee K. Liver abscess caused by Burkholderia pseudomallei in a young man: A case report and review of literature. WORLD J Clin CASES. 2014 Oct 16;2(10):604–7. n.d.

[201] Barman P, Kaur R, Kumar K. Clinically lesser known entity in India: A Report of two cases of Melioidosis. Indian J Crit Care Med Peer-Rev Off Publ Indian Soc Crit Care Med. 2013;17(1):46–8. n.d.

[202] Barman P, Sidhwa H, Shirkhande P. Melioidosis: A Case Report. J Glob Infect Dis. 2011 Apr;3(2):183–6. n.d.

[203] Antony B, Pinto H, Dias M, Shetty AK, Scaria B, Kuruvilla T, et al. Spectrum of melioidosis in the suburbs of Mangalore, S West Coast of India. Southeast Asian J Trop Med Public Health. 2010;41(1):169–74. n.d.

[204] Pandey V, Rao S, Rao S, Acharya K, Chhabra S. Burkholderia pseudomallei musculoskeletal infections (melioidosis) in India. INDIAN J Orthop. 2010 Apr;44(2):216–20. n.d.

[205] Dhodapkar R, Sujatha S, Sivasangeetha K, Prasanth G, Parija SC. Burkholderia pseudomallei infection in a patient with diabetes presenting with multiple splenic abscesses and abscess in the foot: a case report. Cases J. 2008;1(1):224. n.d.

[206] Thomas J, Jayachandran NV, Shenoy Chandrasekhara PK, Lakshmi V, Narsimulu G. Melioidosis--an unusual cause of septic arthritis. Clin Rheumatol. 2008;27:S59-61. n.d.

[207] Mathew S, Perakath B, Mathew G, Sitaram V, Nair A, Lalitha M, et al. Surgical presentation of melioidosis in India. Natl Med J INDIA. 1999 Mar;12(2):59–61. n.d.

[208] Mukhopadhyay C, Chawla K, Krishna S, Nagalakshmi N, Rao SP, Bairy I. Emergence of Burkholderia pseudomallei and pandrug-resistant non-fermenters from southern Karnataka, India. Trans R Soc Trop Med Hyg. 2008;102:S12–7. n.d.

[209] Karthickeyan N, Praveen S, Sunil P, Padmaraj H. Whitmore’s disease: An uncommon urological presentation. Indian J Urol 2014;30:S156.
