# Supplementary material for: Melioidosis in India: A systematic review of individual cases
Source: IJID Reg. 2026 Jan 12;18:100843. doi: 10.1016/j.ijregi.2026.100843 (PMC12874796; doi:10.1016/j.ijregi.2026.100843)
Supplement: Supplementary file 6 [file mmc6.docx]

**Supplementary Table 2:** Joanna Briggs Institute critical appraisal results for included case reports

| **Sn** | **Author** | **Demography** | **History and Presentation** | **Diagnosis** | **Treatment** | **Follow-up** | **Adverse events** |
| --- | --- | --- | --- | --- | --- | --- | --- |
| 1 | Dash 2022 Case 1 | Yes | Yes | Yes | Yes | Yes | Not applicable |
| 2 | Dash 2022 Case 2 | Yes | Yes | Yes | Yes | Yes | Not applicable |
| 3 | Patil 2016 Case 1 | Yes | Yes | No | Yes | Yes | Not applicable |
| 4 | Patil 2016 Case 2 | Yes | No | No | Yes | Yes | Not applicable |
| 5 | Patil 2016 Case 3 | Yes | Yes | Yes | No | Yes | Not applicable |
| 6 | Easow 2024 Case 1 | Yes | Yes | Yes | No | No | Not applicable |
| 7 | Easow 2024 Case 2 | Yes | Yes | Yes | No | Yes | Not applicable |
| 8 | Easow 2024 Case 3 | Yes | Yes | Yes | No | No | Not applicable |
| 9 | Easow 2024 Case 4 | Yes | Yes | Yes | No | No | Not applicable |
| 10 | Easow 2024 Case 5 | Yes | Yes | Yes | No | Yes | Not applicable |
| 11 | Pande 2018 Case 1 | Yes | Yes | Yes | Yes | Yes | Not applicable |
| 12 | Mohanty 2020 Case 1 | Yes | Yes | Yes | Yes | Yes | Not applicable |
| 13 | Mohanty 2020 Case 2 | Yes | Yes | Yes | Yes | Yes | Not applicable |
| 14 | Mohanty 2020 Case 3 | Yes | Yes | Yes | Yes | Yes | Not applicable |
| 15 | Mohanty 2020 Case 4 | Yes | Yes | Yes | Yes | Yes | Not applicable |
| 16 | Mohanty 2020 Case 5 | Yes | Yes | Yes | Yes | Yes | Not applicable |
| 17 | Mohanty 2020 Case 6 | Yes | Yes | Yes | Yes | Yes | Not applicable |
| 18 | Mohanty 2020 Case 7 | Yes | Yes | Yes | No | Yes | Not applicable |
| 19 | Mohanty 2020 Case 8 | Yes | Yes | Yes | No | Yes | Not applicable |
| 20 | Mohanty 2020 Case 9 | Yes | Yes | Yes | Yes | Yes | Not applicable |
| 21 | Varma 2021 Case 1 | Yes | Yes | Yes | Yes | Yes | Not applicable |
| 22 | Mamtora 2018 Case 1 | Yes | Yes | Yes | Yes | Yes | Not applicable |
| 23 | Bojanapati 2024 Case 1 | Yes | Yes | Yes | No | Yes | Not applicable |
| 24 | Singh 2015 Case 1 | Yes | Yes | Yes | Yes | Yes | Not applicable |
| 25 | Yadav 2023 Case 1 | Yes | Yes | Yes | Yes | Yes | Not applicable |
| 26 | Yadav 2023 Case 2 | Yes | Yes | Yes | Yes | Yes | Not applicable |
| 27 | Yadav 2023 Case 3 | Yes | Yes | Yes | Yes | Yes | Not applicable |
| 28 | Yadav 2023 Case 4 | Yes | Yes | Yes | Yes | Yes | Not applicable |
| 29 | Yadav 2023 Case 5 | Yes | Yes | Yes | Yes | Yes | Not applicable |
| 30 | Yadav 2023 Case 6 | Yes | Yes | Yes | No | Yes | Not applicable |
| 31 | Yadav 2023 Case 7 | Yes | Yes | Yes | Yes | Yes | Not applicable |
| 32 | Princess 2017 Case 1 | Yes | Yes | Yes | No | Yes | Not applicable |
| 33 | Shetty 2008 Case 1 | Yes | Yes | Yes | No | Yes | Not applicable |
| 34 | Bhat 2023 Case 1 | Yes | Yes | Yes | Yes | Yes | Not applicable |
| 35 | Garg 2020 Case 1 | Yes | No | No | No | Yes | Not applicable |
| 36 | Garg 2020 Case 2 | Yes | No | No | No | Yes | Not applicable |
| 37 | Garg 2020 Case 3 | Yes | No | Yes | No | Yes | Not applicable |
| 38 | Garg 2020 Case 4 | Yes | No | No | No | Yes | Not applicable |
| 39 | Garg 2020 Case 5 | Yes | No | No | No | Yes | Not applicable |
| 40 | Garg 2020 Case 6 | Yes | No | No | No | Yes | Not applicable |
| 41 | Garg 2020 Case 7 | Yes | No | Yes | No | Yes | Not applicable |
| 42 | Thorve 2023 Case 1 | Yes | Yes | Yes | Yes | Yes | Not applicable |
| 43 | Dubey 2023 Case 1 | Yes | Yes | Yes | Yes | Yes | Not applicable |
| 44 | Dubey 2023 Case 2 | Yes | Yes | Yes | Yes | No | Not applicable |
| 45 | Dubey 2023 Case 3 | Yes | Yes | No | No | Yes | Not applicable |
| 46 | Dubey 2023 Case 4 | Yes | Yes | No | Yes | Yes | Not applicable |
| 47 | Pillai 2014 Case 1 | Yes | Yes | Yes | Yes | Yes | Not applicable |
| 48 | Pillai 2014 Case 2 | Yes | Yes | Yes | No | Yes | Not applicable |
| 49 | Rodrigues 2020 Case 1 | Yes | Yes | No | Yes | Yes | Not applicable |
| 50 | Raji 2018 Case 1 | Yes | Yes | No | No | Yes | Not applicable |
| 51 | Naha 2014 Case 1 | Yes | Yes | No | No | Yes | Not applicable |
| 52 | Viswaroop 2007 Case 1 | Yes | Yes | No | No | Yes | Not applicable |
| 53 | Viswaroop 2007 Case 2 | Yes | Yes | Yes | No | Yes | Not applicable |
| 54 | Vemuri 2021 Case 1 | Yes | Yes | Yes | Yes | Yes | Not applicable |
| 55 | Baikunje 2021 Case 1 | Yes | Yes | Yes | Yes | Yes | Not applicable |
| 56 | Saravu 2015 Case 1 | Yes | Yes | Yes | Yes | Yes | Not applicable |
| 57 | Saravu 2015 Case 2 | Yes | Yes | No | Yes | Yes | Not applicable |
| 58 | Deshmukh 2013 Case 1 | Yes | Yes | Yes | No | Yes | Not applicable |
| 59 | Andraj 2012 Case 1 | Yes | Yes | No | No | Yes | Not applicable |
| 60 | Sampath 2023 Case 1 | Yes | Yes | Yes | Yes | Yes | Not applicable |
| 61 | Singhal 2023 Case 1 | Yes | Yes | No | Yes | Yes | Not applicable |
| 62 | Gupta 2025 Case 1 | Yes | Yes | No | Yes | Yes | Not applicable |
| 63 | Gupta 2025 Case 2 | Yes | Yes | Yes | Yes | Yes | Not applicable |
| 64 | Gupta 2025 Case 3 | Yes | Yes | Yes | Yes | Yes | Not applicable |
| 65 | Gupta 2025 Case 4 | Yes | Yes | No | Yes | Yes | Not applicable |
| 66 | Gupta 2025 Case 5 | Yes | Yes | Yes | Yes | Yes | Not applicable |
| 67 | Gupta 2025 Case 6 | Yes | Yes | Yes | Yes | Yes | Not applicable |
| 68 | Gupta 2025 Case 7 | Yes | Yes | No | Yes | Yes | Not applicable |
| 69 | Gupta 2025 Case 8 | Yes | Yes | Yes | Yes | Yes | Not applicable |
| 70 | Gupta 2025 Case 9 | Yes | Yes | No | Yes | Yes | Not applicable |
| 71 | Subramanian 2020 Case 1 | Yes | Yes | No | Yes | Yes | Not applicable |
| 72 | Subramanian 2020 Case 2 | Yes | Yes | No | Yes | Yes | Not applicable |
| 73 | Subramanian 2020 Case 3 | Yes | Yes | No | No | No | Not applicable |
| 74 | Vidyalakshmi 2008 Case 1 | Yes | Yes | Yes | No | No | Not applicable |
| 75 | Vidyalakshmi 2008 Case 2 | Yes | Yes | No | No | No | Not applicable |
| 76 | Vidyalakshmi 2008 Case 3 | Yes | Yes | No | No | No | Not applicable |
| 77 | Vidyalakshmi 2008 Case 4 | Yes | Yes | No | No | No | Not applicable |
| 78 | Vidyalakshmi 2008 Case 5 | Yes | Yes | No | No | No | Not applicable |
| 79 | Vidyalakshmi 2008 Case 6 | Yes | Yes | No | No | No | Not applicable |
| 80 | Vidyalakshmi 2008 Case 7 | Yes | Yes | No | No | No | Not applicable |
| 81 | Vidyalakshmi 2008 Case 8 | Yes | Yes | Yes | No | No | Not applicable |
| 82 | Vidyalakshmi 2008 Case 9 | Yes | Yes | Yes | No | No | Not applicable |
| 83 | Vidyalakshmi 2008 Case 10 | Yes | Yes | Yes | No | No | Not applicable |
| 84 | Vidyalakshmi 2008 Case 11 | Yes | Yes | No | No | No | Not applicable |
| 85 | Vidyalakshmi 2008 Case 12 | Yes | Yes | No | No | No | Not applicable |
| 86 | Vidyalakshmi 2008 Case 13 | Yes | Yes | No | No | No | Not applicable |
| 87 | Vidyalakshmi 2008 Case 14 | Yes | Yes | No | No | No | Not applicable |
| 88 | Vidyalakshmi 2008 Case 15 | Yes | Yes | No | No | No | Not applicable |
| 89 | Vidyalakshmi 2008 Case 16 | Yes | Yes | No | No | No | Not applicable |
| 90 | Vidyalakshmi 2008 Case 17 | Yes | Yes | No | No | No | Not applicable |
| 91 | Vidyalakshmi 2008 Case 18 | Yes | Yes | No | No | No | Not applicable |
| 92 | Vidyalakshmi 2008 Case 19 | Yes | Yes | No | No | No | Not applicable |
| 93 | Vidyalakshmi 2008 Case 20 | Yes | Yes | No | No | No | Not applicable |
| 94 | Vidyalakshmi 2008 Case 21 | Yes | Yes | No | No | No | Not applicable |
| 95 | Vidyalakshmi 2008 Case 22 | Yes | Yes | No | No | No | Not applicable |
| 96 | Patil 2024 Case 1 | Yes | Yes | Yes | Yes | Yes | Not applicable |
| 97 | Kunthuparambil 2013 Case 1 | Yes | Yes | Yes | Yes | Yes | Not applicable |
| 98 | Rudrabhatla 2022 Case 1 | Yes | Yes | Yes | No | Yes | Not applicable |
| 99 | Ramamoorthi 2013 Case 1 | Yes | Yes | Yes | No | Yes | Not applicable |
| 100 | Ramamoorthi 2013 Case 2 | Yes | Yes | No | Yes | Yes | Not applicable |
| 101 | Ramamoorthi 2013 Case 3 | Yes | Yes | Yes | Yes | Yes | Not applicable |
| 102 | Ramamoorthi 2013 Case 4 | Yes | Yes | No | Yes | Yes | Not applicable |
| 103 | Ramamoorthi 2013 Case 5 | Yes | Yes | Yes | No | Yes | Not applicable |
| 104 | Ramamoorthi 2013 Case 6 | Yes | Yes | Yes | Yes | No | Not applicable |
| 105 | Pradhan 2019 Case 1 | Yes | No | Yes | Yes | Yes | Not applicable |
| 106 | Prabhat 2019 Case 1 | Yes | Yes | Yes | Yes | Yes | Not applicable |
| 107 | Mohan 2021 Case 1 | Yes | Yes | No | Yes | Yes | Not applicable |
| 108 | Mohan 2021 Case 2 | Yes | Yes | Yes | Yes | Yes | Not applicable |
| 109 | James 2013 Case 1 | Yes | Yes | No | Yes | Yes | Not applicable |
| 110 | Mansoor 2016 Case 1 | Yes | Yes | Yes | Yes | Yes | Not applicable |
| 111 | Rajendran 2024 Case 1 | Yes | Yes | No | Yes | Yes | Not applicable |
| 112 | Rajendran 2024 Case 2 | Yes | Yes | No | Yes | Yes | Not applicable |
| 113 | Rajendran 2024 Case 3 | Yes | Yes | No | Yes | Yes | Not applicable |
| 114 | Priyadharshini 2023 Case 1 | Yes | Yes | No | Yes | Yes | Not applicable |
| 115 | Wadwekar 2018 Case 1 | Yes | Yes | Yes | Yes | Yes | Not applicable |
| 116 | Jang 2024 Case 1 | Yes | Yes | Yes | Yes | Yes | Not applicable |
| 117 | Mathew 2020 Case 1 | Yes | Yes | Yes | Yes | No | Not applicable |
| 118 | Teresa 2013 Case 1 | Yes | Yes | Yes | No | Yes | Not applicable |
| 119 | Perumal 2020 Cao 1 | No | Yes | No | Yes | Yes | Not applicable |
| 120 | Perumal 2020 Cao 2 | No | Yes | No | Yes | Yes | Not applicable |
| 121 | Perumal 2020 Cao 3 | No | Yes | No | Yes | Yes | Not applicable |
| 122 | Perumal 2020 Cao 4 | No | Yes | No | Yes | Yes | Not applicable |
| 123 | Perumal 2020 Cao 5 | No | Yes | No | No | Yes | Not applicable |
| 124 | Perumal 2020 Cao 6 | No | Yes | No | Yes | Yes | Not applicable |
| 125 | Perumal 2020 Cao 7 | No | Yes | No | Yes | Yes | Not applicable |
| 126 | Perumal 2020 Cao 8 | No | Yes | Yes | Yes | Yes | Not applicable |
| 127 | Perumal 2020 Cao 9 | No | Yes | No | No | Yes | Not applicable |
| 128 | Perumal 2020 Cao 10 | No | Yes | Yes | Yes | Yes | Not applicable |
| 129 | Perumal 2020 Cao 11 | No | Yes | Yes | Yes | Yes | Not applicable |
| 130 | Perumal 2020 Cao 12 | No | Yes | Yes | Yes | Yes | Not applicable |
| 131 | Perumal 2020 Cao 13 | No | Yes | No | Yes | Yes | Not applicable |
| 132 | Perumal 2020 Cao 14 | No | Yes | No | Yes | Yes | Not applicable |
| 133 | Perumal 2020 Cao 15 | No | Yes | Yes | Yes | Yes | Not applicable |
| 134 | Perumal 2020 Cao 16 | No | Yes | Yes | Yes | Yes | Not applicable |
| 135 | Perumal 2020 Cao 17 | No | Yes | No | Yes | Yes | Not applicable |
| 136 | Perumal 2020 Cao 18 | No | Yes | No | Yes | Yes | Not applicable |
| 137 | Perumal 2020 Cao 19 | No | Yes | Yes | Yes | Yes | Not applicable |
| 138 | Perumal 2020 Cao 20 | No | Yes | Yes | Yes | Yes | Not applicable |
| 139 | Perumal 2020 Cao 21 | No | Yes | Yes | Yes | Yes | Not applicable |
| 140 | Perumal 2020 Cao 22 | No | Yes | Yes | Yes | Yes | Not applicable |
| 141 | Perumal 2020 Cao 23 | No | Yes | Yes | Yes | Yes | Not applicable |
| 142 | Perumal 2020 Cao 24 | No | Yes | Yes | Yes | Yes | Not applicable |
| 143 | Perumal 2020 Cao 25 | No | Yes | No | Yes | Yes | Not applicable |
| 144 | Perumal 2020 Cao 26 | No | Yes | Yes | Yes | Yes | Not applicable |
| 145 | Gautam 2020 Case 1 | Yes | Yes | Yes | No | Yes | Not applicable |
| 146 | Gupta 2021 Case 1 | Yes | Yes | Yes | Yes | Yes | Not applicable |
| 147 | Tripathy 2024 Case 1 | Yes | Yes | Yes | Yes | Yes | Not applicable |
| 148 | Jakribettu 2014 Case 1 | Yes | Yes | Yes | Yes | Yes | Not applicable |
| 149 | Naha 2012 Case 1 | Yes | Yes | No | Yes | Yes | Not applicable |
| 150 | Shobhana 2022 Case 1 | Yes | Yes | No | Yes | Yes | Not applicable |
| 151 | Tirlangi 2025 Case 1 | Yes | No | Yes | Yes | Yes | Not applicable |
| 152 | Veluthat 2021 Case 1 | Yes | Yes | No | Yes | Yes | Not applicable |
| 153 | Sebastian 2024 Case 1 | Yes | Yes | No | Yes | Yes | Not applicable |
| 154 | Patil 2021 Cao 1 | No | Yes | No | Yes | Yes | Not applicable |
| 155 | Agarwal 2023 Case 1 | Yes | Yes | Yes | Yes | Yes | Not applicable |
| 156 | Krishmoorthy 2020 Case 1 | Yes | Yes | Yes | Yes | Yes | Not applicable |
| 157 | D'silva 2018 Case 1 | Yes | Yes | Yes | Yes | Yes | Not applicable |
| 158 | Patro 2019 Case 1 | Yes | Yes | Yes | Yes | Yes | Not applicable |
| 159 | Patro 2019 Case 2 | Yes | Yes | No | Yes | Yes | Not applicable |
| 160 | Jayaprakash 2016 Case 1 | Yes | Yes | No | Yes | Yes | Not applicable |
| 161 | Arockiaraj 2016 Case 1 | Yes | Yes | No | Yes | Yes | Not applicable |
| 162 | Arockiaraj 2016 Case 2 | Yes | Yes | No | Yes | Yes | Not applicable |
| 163 | Arockiaraj 2016 Case 3 | Yes | Yes | No | Yes | Yes | Not applicable |
| 164 | Arockiaraj 2016 Case 4 | Yes | Yes | No | Yes | Yes | Not applicable |
| 165 | Frincy 2020 Case 1 | Yes | Yes | No | No | Yes | Not applicable |
| 166 | Frincy 2020 Case 2 | Yes | Yes | No | Yes | Yes | Not applicable |
| 167 | Jamkhandi 2014 Case 1 | Yes | Yes | Yes | No | Yes | Not applicable |
| 168 | Jamkhandi 2014 Case 2 | Yes | Yes | Yes | No | Yes | Not applicable |
| 169 | Krishna 2024 Case 1 | Yes | Yes | No | Yes | Yes | Not applicable |
| 170 | Subramaniam 2013 Case 1 | Yes | Yes | No | Yes | Yes | Not applicable |
| 171 | Amin 2023 Case 1 | Yes | Yes | Yes | No | No | Not applicable |
| 172 | Amin 2023 Case 2 | Yes | Yes | No | No | No | Not applicable |
| 173 | Amin 2023 Case 3 | Yes | Yes | Yes | No | No | Not applicable |
| 174 | Amin 2023 Case 4 | Yes | Yes | No | No | No | Not applicable |
| 175 | Amin 2023 Case 5 | Yes | Yes | Yes | No | No | Not applicable |
| 176 | Amin 2023 Case 6 | Yes | Yes | Yes | No | Yes | Not applicable |
| 177 | Amin 2023 Case 7 | Yes | Yes | No | No | No | Not applicable |
| 178 | Amin 2023 Case 8 | Yes | Yes | No | No | Yes | Not applicable |
| 179 | Agrawal 2023 Case 1 | Yes | Yes | Yes | No | Yes | Not applicable |
| 180 | Vithiya 2024 Case 1 | Yes | Yes | No | Yes | Yes | Not applicable |
| 181 | Vithiya 2024 Case 2 | Yes | Yes | No | Yes | No | Not applicable |
| 182 | Vithiya 2024 Case 3 | Yes | Yes | No | Yes | Yes | Not applicable |
| 183 | Vithiya 2024 Case 4 | Yes | No | No | Yes | Yes | Not applicable |
| 184 | Vithiya 2024 Case 5 | Yes | Yes | No | Yes | Yes | Not applicable |
| 185 | Vithiya 2024 Case 6 | Yes | Yes | Yes | No | Yes | Not applicable |
| 186 | Vithiya 2024 Case 7 | Yes | No | No | No | No | Not applicable |
| 187 | Vithiya 2024 Case 8 | Yes | Yes | Yes | No | Yes | Not applicable |
| 188 | Vithiya 2024 Case 9 | Yes | Yes | Yes | No | No | Not applicable |
| 189 | Vithiya 2024 Case 10 | Yes | Yes | Yes | No | Yes | Not applicable |
| 190 | Vithiya 2024 Case 11 | Yes | Yes | No | No | Yes | Not applicable |
| 191 | Vithiya 2024 Cao 12 | No | Yes | Yes | Yes | Yes | Not applicable |
| 192 | Vithiya 2024 Case 13 | Yes | Yes | Yes | Yes | Yes | Not applicable |
| 193 | Vithiya 2024 Case 14 | Yes | Yes | Yes | Yes | Yes | Not applicable |
| 194 | Vithiya 2024 Case 15 | Yes | Yes | Yes | Yes | Yes | Not applicable |
| 195 | Vithiya 2024 Case 16 | Yes | Yes | Yes | Yes | No | Not applicable |
| 196 | Vithiya 2024 Case 17 | Yes | Yes | Yes | Yes | Yes | Not applicable |
| 197 | Vithiya 2024 Case 18 | Yes | Yes | Yes | Yes | Yes | Not applicable |
| 198 | Vithiya 2024 Case 19 | Yes | Yes | Yes | Yes | Yes | Not applicable |
| 199 | Vithiya 2024 Case 20 | Yes | Yes | No | Yes | Yes | Not applicable |
| 200 | Vithiya 2024 Case 21 | Yes | Yes | Yes | Yes | Yes | Not applicable |
| 201 | Vithiya 2024 Case 22 | Yes | Yes | No | No | No | Not applicable |
| 202 | Nair 2018 Case 1 | Yes | Yes | No | Yes | Yes | Not applicable |
| 203 | Bhandary 2018 Case 1 | Yes | Yes | No | No | Yes | Not applicable |
| 204 | Devi 2024 Case 1 | Yes | Yes | Yes | Yes | Yes | Not applicable |
| 205 | Vithiya 2024 Case 1 | Yes | Yes | Yes | Yes | Yes | Not applicable |
| 206 | Vithiya 2024 Case 2 | Yes | Yes | Yes | No | No | Not applicable |
| 207 | Vithiya 2024 Case 3 | Yes | Yes | Yes | No | Yes | Not applicable |
| 208 | Vithiya 2024 Case 4 | Yes | Yes | No | Yes | Yes | Not applicable |
| 209 | Vithiya 2024 Case 5 | Yes | Yes | Yes | Yes | Yes | Not applicable |
| 210 | Vithiya 2024 Case 6 | Yes | Yes | Yes | Yes | Yes | Not applicable |
| 211 | Vithiya 2024 Case 7 | Yes | Yes | Yes | Yes | Yes | Not applicable |
| 212 | Vithiya 2024 Case 8 | Yes | Yes | Yes | Yes | Yes | Not applicable |
| 213 | Vithiya 2024 Case 9 | Yes | Yes | Yes | Yes | No | Not applicable |
| 214 | Vithiya 2024 Cao 10 | No | Yes | Yes | Yes | Yes | Not applicable |
| 215 | Vithiya 2024 Case 11 | Yes | Yes | No | No | Yes | Not applicable |
| 216 | Vithiya 2024 Case 12 | Yes | Yes | No | Yes | Yes | Not applicable |
| 217 | Vithiya 2024 Case 13 | Yes | Yes | Yes | Yes | Yes | Not applicable |
| 218 | Jacob 2018 Case 1 | Yes | Yes | Yes | No | Yes | Not applicable |
| 219 | Mukhopadhyay 2010 Case 1 | Yes | Yes | Yes | No | Yes | Not applicable |
| 220 | Mukhopadhyay 2010 Case 2 | Yes | Yes | Yes | Yes | Yes | Not applicable |
| 221 | Mahapatra 2021 Cao 1 | No | Yes | No | Yes | Yes | Not applicable |
| 222 | Nivedhana 2016 Case 1 | Yes | Yes | Yes | No | Yes | Not applicable |
| 223 | Shamim 2016 Case 1 | Yes | Yes | No | Yes | Yes | Not applicable |
| 224 | Shamim 2016 Case 2 | Yes | Yes | No | No | Yes | Not applicable |
| 225 | Shetty 2022 Case 1 | Yes | Yes | Yes | No | Yes | Not applicable |
| 226 | Nayak 2018 Case 1 | Yes | Yes | Yes | Yes | Yes | Not applicable |
| 227 | Kumar 2024 Case 1 | Yes | Yes | Yes | Yes | Yes | Not applicable |
| 228 | Kumar 2024 Case 2 | Yes | Yes | Yes | Yes | Yes | Not applicable |
| 229 | Kumar 2024 Case 3 | Yes | No | Yes | Yes | Yes | Not applicable |
| 230 | Kumar 2024 Case 4 | Yes | No | Yes | Yes | Yes | Not applicable |
| 231 | Kumar 2024 Case 5 | Yes | Yes | Yes | Yes | Yes | Not applicable |
| 232 | Kumar 2024 Case 6 | Yes | Yes | Yes | Yes | Yes | Not applicable |
| 233 | Chowdhury 2024 Case 1 | Yes | Yes | Yes | No | Yes | Not applicable |
| 234 | Rajinikanth 2008 Case 1 | Yes | Yes | No | Yes | No | Not applicable |
| 235 | Jabeen 2021 Case 1 | Yes | Yes | No | Yes | Yes | Not applicable |
| 236 | Kumari 2024 Case 1 | Yes | Yes | No | Yes | Yes | Not applicable |
| 237 | Kumari 2024 Case 2 | Yes | Yes | Yes | Yes | No | Not applicable |
| 238 | Kumari 2024 Case 3 | Yes | Yes | Yes | Yes | Yes | Not applicable |
| 239 | Gupta 2022 Case 1 | Yes | Yes | Yes | No | Yes | Not applicable |
| 240 | Gupta 2022 Case 2 | Yes | No | Yes | No | Yes | Not applicable |
| 241 | Sovane 2024 Case 1 | Yes | Yes | Yes | Yes | Yes | Not applicable |
| 242 | Indulekha 2022 Case 1 | Yes | Yes | Yes | No | Yes | Not applicable |
| 243 | Ekka 2017 Case 1 | Yes | Yes | No | Yes | Yes | Not applicable |
| 244 | Mohapatra 2024 Case 1 | Yes | Yes | Yes | Yes | Yes | Not applicable |
| 245 | Sivaselvi 2024 Case 1 | Yes | Yes | Yes | No | Yes | Not applicable |
| 246 | Khatod 2024 Case 1 | Yes | Yes | Yes | Yes | Yes | Not applicable |
| 247 | Uddandam 2023 Case 1 | Yes | Yes | Yes | Yes | Yes | Not applicable |
| 248 | Jain 2023 Case 1 | Yes | Yes | No | Yes | Yes | Not applicable |
| 249 | Prasanna 2023 Case 1 | Yes | No | Yes | No | Yes | Not applicable |
| 250 | Prasanna 2023 Case 2 | Yes | Yes | No | No | Yes | Not applicable |
| 251 | Prasanna 2023 Case 3 | Yes | Yes | No | No | Yes | Not applicable |
| 252 | Meenakumari 2023 Case 1 | Yes | Yes | Yes | Yes | Yes | Not applicable |
| 253 | Naik 2023 Case 1 | Yes | Yes | Yes | No | Yes | Not applicable |
| 254 | Naik 2023 Case 2 | Yes | Yes | Yes | No | Yes | Not applicable |
| 255 | Naik 2023 Case 3 | Yes | Yes | Yes | No | Yes | Not applicable |
| 256 | Naik 2023 Case 4 | Yes | Yes | Yes | No | Yes | Not applicable |
| 257 | Naik 2023 Case 5 | Yes | Yes | Yes | No | Yes | Not applicable |
| 258 | Naik 2023 Case 6 | Yes | Yes | No | No | Yes | Not applicable |
| 259 | Kumar 2023 Case 1 | Yes | Yes | No | Yes | Yes | Not applicable |
| 260 | Sharma 2022 Case 1 | Yes | Yes | Yes | Yes | Yes | Not applicable |
| 261 | Sharma 2022 Case 2 | Yes | Yes | Yes | Yes | Yes | Not applicable |
| 262 | Sharma 2022 Case 3 | Yes | Yes | Yes | Yes | Yes | Not applicable |
| 263 | Bhaskaran 2022 Case 1 | Yes | Yes | Yes | No | Yes | Not applicable |
| 264 | Bhaskaran 2022 Case 2 | Yes | Yes | No | Yes | Yes | Not applicable |
| 265 | Agrawal 2022 Case 1 | Yes | Yes | Yes | Yes | Yes | Not applicable |
| 266 | Chandra 2022 Case 1 | Yes | Yes | Yes | No | Yes | Not applicable |
| 267 | Shanmugam 2021 Case 1 | Yes | Yes | No | No | Yes | Not applicable |
| 268 | Radhakrishnan 2021 Cao 1 | No | No | Yes | No | Yes | Not applicable |
| 269 | Radhakrishnan 2021 Cao 2 | No | No | Yes | No | Yes | Not applicable |
| 270 | Radhakrishnan 2021 Cao 3 | No | No | Yes | No | Yes | Not applicable |
| 271 | Radhakrishnan 2021 Cao 4 | No | No | Yes | No | Yes | Not applicable |
| 272 | Radhakrishnan 2021 Cao 5 | No | No | Yes | No | Yes | Not applicable |
| 273 | Radhakrishnan 2021 Cao 6 | No | No | Yes | No | Yes | Not applicable |
| 274 | Radhakrishnan 2021 Cao 7 | No | No | Yes | No | No | Not applicable |
| 275 | Radhakrishnan 2021 Cao 8 | No | No | Yes | No | Yes | Not applicable |
| 276 | Radhakrishnan 2021 Cao 9 | No | No | Yes | No | Yes | Not applicable |
| 277 | Radhakrishnan 2021 Cao 10 | No | No | Yes | No | Yes | Not applicable |
| 278 | Radhakrishnan 2021 Cao 11 | No | No | Yes | No | Yes | Not applicable |
| 279 | Radhakrishnan 2021 Cao 12 | No | No | Yes | No | Yes | Not applicable |
| 280 | Radhakrishnan 2021 Cao 13 | No | No | Yes | No | Yes | Not applicable |
| 281 | Radhakrishnan 2021 Cao 14 | No | No | Yes | No | No | Not applicable |
| 282 | Radhakrishnan 2021 Cao 15 | No | No | Yes | No | No | Not applicable |
| 283 | Radhakrishnan 2021 Cao 16 | No | No | Yes | No | Yes | Not applicable |
| 284 | Radhakrishnan 2021 Cao 17 | No | No | Yes | No | Yes | Not applicable |
| 285 | Radhakrishnan 2021 Cao 18 | No | No | Yes | No | Yes | Not applicable |
| 286 | Radhakrishnan 2021 Cao 19 | No | No | Yes | No | Yes | Not applicable |
| 287 | Radhakrishnan 2021 Cao 20 | No | No | Yes | No | Yes | Not applicable |
| 288 | Radhakrishnan 2021 Cao 21 | No | No | Yes | No | Yes | Not applicable |
| 289 | Radhakrishnan 2021 Cao 22 | No | No | Yes | No | Yes | Not applicable |
| 290 | Radhakrishnan 2021 Cao 23 | No | No | Yes | No | Yes | Not applicable |
| 291 | Radhakrishnan 2021 Cao 24 | No | No | Yes | No | Yes | Not applicable |
| 292 | Radhakrishnan 2021 Cao 25 | No | No | Yes | No | Yes | Not applicable |
| 293 | Radhakrishnan 2021 Cao 26 | No | No | Yes | No | Yes | Not applicable |
| 294 | Radhakrishnan 2021 Cao 27 | No | No | Yes | No | Yes | Not applicable |
| 295 | Radhakrishnan 2021 Cao 28 | No | No | Yes | No | No | Not applicable |
| 296 | Radhakrishnan 2021 Cao 29 | No | No | Yes | No | Yes | Not applicable |
| 297 | Radhakrishnan 2021 Cao 30 | No | No | Yes | No | Yes | Not applicable |
| 298 | Radhakrishnan 2021 Cao 31 | No | No | Yes | No | Yes | Not applicable |
| 299 | Radhakrishnan 2021 Cao 32 | No | No | Yes | No | Yes | Not applicable |
| 300 | Radhakrishnan 2021 Cao 33 | No | No | Yes | No | Yes | Not applicable |
| 301 | Sundaram 2021 Case 1 | Yes | Yes | Yes | No | Yes | Not applicable |
| 302 | Muthina 2021 Case 1 | Yes | Yes | Yes | Yes | Yes | Not applicable |
| 303 | Iyer 2021 Case 1 | Yes | Yes | Yes | Yes | Yes | Not applicable |
| 304 | Mishra 2021 Case 1 | Yes | Yes | Yes | Yes | Yes | Not applicable |
| 305 | Chatterjee 2021 Case 1 | Yes | No | Yes | No | Yes | Not applicable |
| 306 | Chatterjee 2021 Case 2 | Yes | Yes | No | Yes | Yes | Not applicable |
| 307 | Gupta 2021 Case 1 | Yes | Yes | Yes | Yes | Yes | Not applicable |
| 308 | Gupta 2021 Case 2 | Yes | Yes | Yes | Yes | Yes | Not applicable |
| 309 | Gupta 2021 Case 3 | Yes | Yes | Yes | Yes | Yes | Not applicable |
| 310 | Gupta 2021 Case 4 | Yes | Yes | Yes | Yes | Yes | Not applicable |
| 311 | Gupta 2021 Case 5 | Yes | Yes | Yes | Yes | Yes | Not applicable |
| 312 | Gupta 2021 Case 6 | Yes | Yes | Yes | Yes | No | Not applicable |
| 313 | Gupta 2021 Case 7 | Yes | Yes | Yes | Yes | Yes | Not applicable |
| 314 | Gupta 2021 Case 8 | Yes | Yes | Yes | Yes | Yes | Not applicable |
| 315 | Gupta 2021 Case 9 | Yes | Yes | Yes | Yes | Yes | Not applicable |
| 316 | Gupta 2021 Case 10 | Yes | Yes | Yes | Yes | Yes | Not applicable |
| 317 | Gupta 2021 Case 11 | Yes | Yes | Yes | Yes | Yes | Not applicable |
| 318 | Kulkarni 2020 Case 1 | Yes | Yes | Yes | No | No | Not applicable |
| 319 | Kulkarni 2020 Case 2 | Yes | Yes | No | No | No | Not applicable |
| 320 | Rao 2020 Cao 1 | No | Yes | Yes | Yes | Yes | Not applicable |
| 321 | Rahman 2020 Case 1 | Yes | Yes | Yes | Yes | Yes | Not applicable |
| 322 | Rahman 2020 Case 2 | Yes | Yes | Yes | Yes | Yes | Not applicable |
| 323 | Koshy 2019 Case 1 | Yes | Yes | Yes | Yes | Yes | Not applicable |
| 324 | Koshy 2019 Case 2 | Yes | Yes | Yes | Yes | Yes | Not applicable |
| 325 | Koshy 2019 Case 3 | Yes | Yes | Yes | Yes | Yes | Not applicable |
| 326 | Koshy 2019 Case 4 | Yes | Yes | Yes | Yes | Yes | Not applicable |
| 327 | Koshy 2019 Case 5 | Yes | Yes | Yes | Yes | Yes | Not applicable |
| 328 | Koshy 2019 Case 6 | Yes | Yes | Yes | Yes | Yes | Not applicable |
| 329 | Koshy 2019 Case 7 | Yes | Yes | Yes | Yes | Yes | Not applicable |
| 330 | Koshy 2019 Case 8 | Yes | Yes | Yes | Yes | Yes | Not applicable |
| 331 | Koshy 2019 Case 9 | Yes | Yes | Yes | Yes | Yes | Not applicable |
| 332 | Koshy 2019 Case 10 | Yes | Yes | Yes | Yes | Yes | Not applicable |
| 333 | Koshy 2019 Case 11 | Yes | Yes | Yes | Yes | Yes | Not applicable |
| 334 | Koshy 2019 Case 12 | Yes | Yes | Yes | Yes | Yes | Not applicable |
| 335 | Koshy 2019 Case 13 | Yes | Yes | Yes | Yes | Yes | Not applicable |
| 336 | Koshy 2019 Case 14 | Yes | Yes | Yes | Yes | Yes | Not applicable |
| 337 | Koshy 2019 Case 15 | Yes | Yes | Yes | Yes | Yes | Not applicable |
| 338 | Koshy 2019 Case 16 | Yes | Yes | Yes | Yes | Yes | Not applicable |
| 339 | Koshy 2019 Case 17 | Yes | Yes | Yes | Yes | Yes | Not applicable |
| 340 | Koshy 2019 Case 18 | Yes | Yes | Yes | Yes | Yes | Not applicable |
| 341 | Koshy 2019 Case 19 | Yes | Yes | Yes | Yes | Yes | Not applicable |
| 342 | Koshy 2019 Case 20 | Yes | Yes | Yes | Yes | Yes | Not applicable |
| 343 | Annamalai 2019 Case 1 | Yes | Yes | Yes | Yes | Yes | Not applicable |
| 344 | Gunasekaran 2018 Case 1 | Yes | Yes | Yes | No | Yes | Not applicable |
| 345 | Kamath 2018 Case 1 | Yes | Yes | No | Yes | Yes | Not applicable |
| 346 | Garg 2018 Case 1 | Yes | Yes | Yes | Yes | Yes | Not applicable |
| 347 | Nair 2017 Case 1 | Yes | Yes | No | Yes | Yes | Not applicable |
| 348 | Patil 2017 Case 1 | Yes | Yes | Yes | Yes | Yes | Not applicable |
| 349 | Antony 2017 Case 1 | Yes | Yes | Yes | Yes | Yes | Not applicable |
| 350 | Kundangar 2017 Case 1 | Yes | Yes | Yes | Yes | Yes | Not applicable |
| 351 | Achappa 2016 Case 1 | Yes | Yes | No | Yes | Yes | Not applicable |
| 352 | Sathiavageesan 2016 Case 1 | Yes | Yes | Yes | Yes | Yes | Not applicable |
| 353 | Madi 2016 Case 1 | Yes | Yes | Yes | Yes | Yes | Not applicable |
| 354 | Ray 2016 Case 1 | Yes | Yes | Yes | Yes | Yes | Not applicable |
| 355 | Ray 2016 Case 2 | Yes | Yes | Yes | Yes | Yes | Not applicable |
| 356 | Ray 2016 Case 3 | Yes | Yes | Yes | Yes | Yes | Not applicable |
| 357 | Ray 2016 Case 4 | Yes | Yes | Yes | Yes | Yes | Not applicable |
| 358 | Ray 2016 Case 5 | Yes | Yes | Yes | Yes | Yes | Not applicable |
| 359 | Ray 2016 Case 6 | Yes | Yes | Yes | Yes | Yes | Not applicable |
| 360 | Ray 2016 Case 7 | Yes | No | Yes | Yes | Yes | Not applicable |
| 361 | Ray 2016 Case 8 | Yes | Yes | Yes | Yes | Yes | Not applicable |
| 362 | Vijaykumar 2016 Case 1 | Yes | Yes | Yes | No | Yes | Not applicable |
| 363 | Amrutha 2016 Case 1 | Yes | No | Yes | Yes | Yes | Not applicable |
| 364 | Mohanty 2016 Case 1 | Yes | Yes | Yes | Yes | Yes | Not applicable |
| 365 | Vaid 2015 Case 1 | Yes | Yes | Yes | Yes | Yes | Not applicable |
| 366 | Garg 2015 Case 1 | Yes | Yes | No | Yes | Yes | Not applicable |
| 367 | Garg 2015 Case 2 | Yes | Yes | No | Yes | Yes | Not applicable |
| 368 | Padmaja 2015 Case 1 | Yes | No | Yes | Yes | Yes | Not applicable |
| 369 | Padmaja 2015 Case 2 | Yes | Yes | Yes | No | Yes | Not applicable |
| 370 | Shetty 2015 Case 1 | Yes | Yes | Yes | Yes | Yes | Not applicable |
| 371 | Neliyathodi 2015 Case 1 | Yes | Yes | No | Yes | Yes | Not applicable |
| 372 | Sood 2014 Cao 1 | No | Yes | Yes | Yes | Yes | Not applicable |
| 373 | Totagi 2014 Case 1 | Yes | Yes | Yes | No | Yes | Not applicable |
| 374 | Totagi 2014 Case 2 | Yes | Yes | Yes | No | Yes | Not applicable |
| 375 | Subramanyam 2014 Case 1 | Yes | No | Yes | No | Yes | Not applicable |
| 376 | Karthickeyan 2014 Cao 1 | No | Yes | Yes | Yes | Yes | Not applicable |
| 377 | Krovvidi 2013 Case 1 | Yes | Yes | Yes | No | Yes | Not applicable |
| 378 | Esther 2013 Case 1 | Yes | Yes | Yes | Yes | Yes | Not applicable |
| 379 | Boruah 2013 Case 1 | Yes | Yes | Yes | No | Yes | Not applicable |
| 380 | Bhat 2012 Cao 1 | No | Yes | Yes | Yes | Yes | Not applicable |
| 381 | Prasad 2012 Case 1 | Yes | Yes | No | Yes | Yes | Not applicable |
| 382 | Rajadhyaksha 2012 Case 1 | Yes | Yes | No | Yes | Yes | Not applicable |
| 383 | Behera 2012 Case 1 | Yes | Yes | Yes | No | Yes | Not applicable |
| 384 | Saravu 2012 Case 1 | Yes | Yes | Yes | Yes | Yes | Not applicable |
| 385 | Viswanathan 2012 Case 1 | Yes | Yes | No | Yes | Yes | Not applicable |
| 386 | Shivbalan 2010 Case 1 | Yes | Yes | Yes | Yes | Yes | Not applicable |
| 387 | Mukhopadhyay 2010 Case 1 | Yes | Yes | Yes | Yes | Yes | Not applicable |
| 388 | Mukhopadhyay 2010 Case 2 | Yes | Yes | Yes | Yes | Yes | Not applicable |
| 389 | Kulkarni 2010 Case 1 | Yes | Yes | Yes | No | Yes | Not applicable |
| 390 | Shetty 2010 Case 1 | Yes | Yes | Yes | Yes | Yes | Not applicable |
| 391 | Kuruvilla 2010 Case 1 | Yes | Yes | Yes | No | Yes | Not applicable |
| 392 | Shenoy 2009 Case 1 | Yes | Yes | No | No | Yes | Not applicable |
| 393 | Valsalan 2009 Case 1 | Yes | Yes | No | Yes | Yes | Not applicable |
| 394 | Valsalan 2009 Case 2 | Yes | Yes | Yes | Yes | Yes | Not applicable |
| 395 | Ray 2009 Case 1 | Yes | Yes | Yes | Yes | Yes | Not applicable |
| 396 | Noyal 2009 Case 1 | Yes | No | Yes | Yes | Yes | Not applicable |
| 397 | Saravu 2008 Case 1 | Yes | Yes | Yes | No | Yes | Not applicable |
| 398 | Saravu 2008 Case 1 | Yes | Yes | Yes | Yes | Yes | Not applicable |
| 399 | Saravu 2008 Case 2 | Yes | Yes | No | No | Yes | Not applicable |
| 400 | Saravu 2008 Case 3 | Yes | Yes | No | No | Yes | Not applicable |
| 401 | Saravu 2008 Case 4 | Yes | Yes | Yes | No | Yes | Not applicable |
| 402 | Saravu 2008 Case 5 | Yes | No | No | No | No | Not applicable |
| 403 | Saravu 2008 Case 6 | Yes | Yes | Yes | Yes | Yes | Not applicable |
| 404 | Valsalan 2008 Case 1 | Yes | Yes | Yes | Yes | Yes | Not applicable |
| 405 | Kumar 2008 Cao 1 | No | No | Yes | No | Yes | Not applicable |
| 406 | Kumar 2008 Cao 2 | No | No | No | Yes | Yes | Not applicable |
| 407 | Kumar 2008 Cao 3 | No | No | No | Yes | Yes | Not applicable |
| 408 | Kumar 2008 Cao 4 | No | No | No | Yes | No | Not applicable |
| 409 | Kumar 2008 Cao 5 | No | No | No | Yes | Yes | Not applicable |
| 410 | Kumar 2008 Cao 6 | No | No | No | Yes | Yes | Not applicable |
| 411 | Mukhopadhyay 2007 Case 1 | Yes | Yes | Yes | Yes | Yes | Not applicable |
| 412 | Mukhopadhyay 2007 Case 2 | Yes | Yes | Yes | Yes | Yes | Not applicable |
| 413 | Dias 2004 Case 1 | Yes | No | Yes | No | Yes | Not applicable |
| 414 | Anuradha 2003 Case 1 | Yes | Yes | Yes | No | Yes | Not applicable |
| 415 | Rao 2002 Case 1 | Yes | No | Yes | Yes | Yes | Not applicable |
| 416 | Rao 2002 Case 2 | Yes | No | Yes | Yes | Yes | Not applicable |
| 417 | Rao 2002 Case 3 | Yes | No | Yes | Yes | Yes | Not applicable |
| 418 | Rao 2002 Case 4 | Yes | No | Yes | Yes | Yes | Not applicable |
| 419 | Rao 2002 Case 5 | Yes | No | Yes | Yes | Yes | Not applicable |
| 420 | Rao 1999 Case 1 | Yes | Yes | No | No | Yes | Not applicable |
| 421 | Lath 1998 Case 1 | Yes | Yes | Yes | No | Yes | Not applicable |
| 422 | Sanklecha 1997 Case 1 | Yes | Yes | No | No | Yes | Not applicable |
| 423 | Sanklecha 1997 Case 2 | Yes | Yes | Yes | Yes | Yes | Not applicable |
| 424 | Cherian 1996 Case 1 | Yes | Yes | Yes | Yes | Yes | Not applicable |
| 425 | Raghavan 1991 Case 1 | Yes | Yes | Yes | Yes | Yes | Not applicable |
| 426 | Muthanikkatt 2025 Case 1 | Yes | Yes | Yes | No | Yes | Not applicable |
| 427 | Gupta 2024 Case 1 | Yes | Yes | Yes | No | Yes | Not applicable |
| 428 | Gupta 2024 Case 2 | Yes | Yes | Yes | No | Yes | Not applicable |
| 429 | Gupta 2024 Case 3 | Yes | Yes | Yes | No | Yes | Not applicable |
| 430 | Gupta 2024 Case 4 | Yes | Yes | Yes | No | Yes | Not applicable |
| 431 | Devi 2025 Case 1 | Yes | Yes | Yes | Yes | Yes | Not applicable |
| 432 | Gupta 2024 Case 1 | Yes | Yes | Yes | No | Yes | Not applicable |
| 433 | Gupta 2024 Case 2 | Yes | Yes | Yes | No | Yes | Not applicable |
| 434 | Gupta 2024 Case 3 | Yes | Yes | Yes | No | Yes | Not applicable |
| 435 | Gupta 2024 Case 4 | Yes | Yes | Yes | No | Yes | Not applicable |
| 436 | Gupta 2024 Case 5 | Yes | Yes | Yes | No | Yes | Not applicable |
| 437 | Gupta 2024 Case 6 | Yes | Yes | Yes | No | Yes | Not applicable |
| 438 | Gupta 2024 Case 7 | Yes | Yes | Yes | No | Yes | Not applicable |
| 439 | Gupta 2024 Case 8 | Yes | Yes | Yes | No | Yes | Not applicable |
| 440 | Waseem 2023 Case 1 | Yes | Yes | Yes | Yes | Yes | Not applicable |
| 441 | Bhaskar 2023 Case 1 | Yes | Yes | Yes | Yes | Yes | Not applicable |
| 442 | Harsha 2023 Case 1 | Yes | Yes | Yes | Yes | Yes | Not applicable |
| 443 | Jayakumar 2022 Case 1 | Yes | Yes | Yes | Yes | Yes | Not applicable |
| 444 | Agrawal 2023 Cao 1 | No | Yes | Yes | Yes | Yes | Not applicable |
| 445 | Singhal 2022 Case 1 | Yes | Yes | Yes | No | Yes | Not applicable |
| 446 | Bahuleyan 2022 Case 1 | Yes | Yes | Yes | Yes | Yes | Not applicable |
| 447 | Singhai 2022 Case 1 | Yes | Yes | Yes | Yes | Yes | Not applicable |
| 448 | Deb 2021 Case 1 | Yes | Yes | Yes | Yes | Yes | Not applicable |
| 449 | Mohanty 2022 Case 1 | Yes | Yes | No | Yes | Yes | Not applicable |
| 450 | Halim 2021 Case 1 | Yes | Yes | Yes | Yes | Yes | Not applicable |
| 451 | Nair 2021 Case 1 | Yes | Yes | Yes | Yes | Yes | Not applicable |
| 452 | Patel 2021 Case 1 | Yes | Yes | Yes | No | Yes | Not applicable |
| 453 | Patel 2021 Case 2 | Yes | Yes | No | No | Yes | Not applicable |
| 454 | Patel 2021 Case 3 | Yes | Yes | Yes | No | Yes | Not applicable |
| 455 | Patel 2021 Case 4 | Yes | Yes | No | No | Yes | Not applicable |
| 456 | Patel 2021 Case 5 | Yes | Yes | Yes | No | Yes | Not applicable |
| 457 | Patel 2021 Case 6 | Yes | Yes | Yes | No | Yes | Not applicable |
| 458 | Patel 2021 Case 7 | Yes | Yes | Yes | No | Yes | Not applicable |
| 459 | Patel 2021 Case 8 | Yes | Yes | No | No | Yes | Not applicable |
| 460 | Patel 2021 Case 9 | Yes | Yes | Yes | No | Yes | Not applicable |
| 461 | Patel 2021 Case 10 | Yes | Yes | No | No | Yes | Not applicable |
| 462 | Patel 2021 Case 11 | Yes | Yes | No | No | Yes | Not applicable |
| 463 | Maramattom 2021 Case 1 | Yes | Yes | Yes | Yes | Yes | Not applicable |
| 464 | Savaj 2020 Case 1 | Yes | Yes | Yes | No | Yes | Not applicable |
| 465 | Kandhasamy 2020 Case 1 | Yes | Yes | Yes | Yes | Yes | Not applicable |
| 466 | Parija 2020 Case 1 | Yes | Yes | No | Yes | Yes | Not applicable |
| 467 | Subramony 2019 Case 1 | Yes | Yes | Yes | Yes | Yes | Not applicable |
| 468 | Alexander 2018 Case 1 | Yes | Yes | Yes | Yes | Yes | Not applicable |
| 469 | Saluja 2019 Case 1 | Yes | Yes | Yes | Yes | Yes | Not applicable |
| 470 | Ninan 2018 Case 1 | Yes | No | Yes | Yes | Yes | Not applicable |
| 471 | Gouse 2017 Case 1 | Yes | Yes | Yes | Yes | Yes | Not applicable |
| 472 | Gouse 2017 Case 2 | Yes | Yes | Yes | Yes | Yes | Not applicable |
| 473 | Gouse 2017 Case 3 | Yes | Yes | Yes | Yes | Yes | Not applicable |
| 474 | Gouse 2017 Case 4 | Yes | Yes | Yes | Yes | Yes | Not applicable |
| 475 | Gouse 2017 Case 5 | Yes | Yes | Yes | Yes | Yes | Not applicable |
| 476 | Gouse 2017 Case 6 | Yes | Yes | Yes | Yes | Yes | Not applicable |
| 477 | Gouse 2017 Case 7 | Yes | Yes | Yes | Yes | Yes | Not applicable |
| 478 | Gouse 2017 Case 8 | Yes | Yes | Yes | Yes | Yes | Not applicable |
| 479 | Gouse 2017 Case 9 | Yes | Yes | Yes | Yes | Yes | Not applicable |
| 480 | Gouse 2017 Case 10 | Yes | Yes | Yes | Yes | Yes | Not applicable |
| 481 | Gouse 2017 Case 11 | Yes | Yes | Yes | Yes | Yes | Not applicable |
| 482 | Gouse 2017 Case 12 | Yes | Yes | Yes | Yes | Yes | Not applicable |
| 483 | Gouse 2017 Case 13 | Yes | Yes | Yes | Yes | Yes | Not applicable |
| 484 | Gouse 2017 Case 14 | Yes | Yes | Yes | Yes | Yes | Not applicable |
| 485 | Gouse 2017 Case 15 | Yes | Yes | Yes | Yes | Yes | Not applicable |
| 486 | Gouse 2017 Case 16 | Yes | Yes | Yes | Yes | Yes | Not applicable |
| 487 | Gouse 2017 Case 17 | Yes | Yes | Yes | Yes | Yes | Not applicable |
| 488 | Gouse 2017 Case 18 | Yes | Yes | Yes | Yes | Yes | Not applicable |
| 489 | Jagtap 2017 Case 1 | Yes | Yes | Yes | Yes | Yes | Not applicable |
| 490 | Jagtap 2017 Case 2 | Yes | Yes | Yes | Yes | Yes | Not applicable |
| 491 | Jagtap 2017 Case 3 | Yes | Yes | Yes | Yes | Yes | Not applicable |
| 492 | Jagtap 2017 Case 4 | Yes | Yes | Yes | Yes | Yes | Not applicable |
| 493 | Jagtap 2017 Case 5 | Yes | Yes | Yes | Yes | Yes | Not applicable |
| 494 | Jagtap 2017 Case 6 | Yes | Yes | Yes | Yes | Yes | Not applicable |
| 495 | Jagtap 2017 Case 7 | Yes | Yes | Yes | Yes | Yes | Not applicable |
| 496 | Jagtap 2017 Case 8 | Yes | Yes | Yes | Yes | Yes | Not applicable |
| 497 | Jagtap 2017 Case 9 | Yes | Yes | Yes | Yes | No | Not applicable |
| 498 | Goel 2016 Case 1 | Yes | Yes | Yes | Yes | Yes | Not applicable |
| 499 | Kumar 2017 Case 1 | Yes | Yes | Yes | Yes | Yes | Not applicable |
| 500 | Karuna 2015 Case 1 | Yes | Yes | No | Yes | Yes | Not applicable |
| 501 | Tyagi 2014 Case 1 | Yes | Yes | Yes | Yes | Yes | Not applicable |
| 502 | Pal 2014 Case 1 | Yes | Yes | No | Yes | Yes | Not applicable |
| 503 | Barman 2013 Case 2 | Yes | No | Yes | No | Yes | Not applicable |
| 504 | Barman 2011 Case 1 | Yes | Yes | Yes | Yes | Yes | Not applicable |
| 505 | Antony 2010 Case 1 | Yes | Yes | Yes | Yes | Yes | Not applicable |
| 506 | Antony 2010 Case 2 | Yes | Yes | Yes | No | Yes | Not applicable |
| 507 | Antony 2010 Case 3 | Yes | Yes | Yes | No | Yes | Not applicable |
| 508 | Antony 2010 Case 4 | Yes | Yes | Yes | No | Yes | Not applicable |
| 509 | Antony 2010 Case 5 | Yes | Yes | Yes | No | Yes | Not applicable |
| 510 | Antony 2010 Case 6 | Yes | Yes | Yes | No | Yes | Not applicable |
| 511 | Antony 2010 Case 7 | Yes | Yes | Yes | No | Yes | Not applicable |
| 512 | Antony 2010 Case 8 | Yes | Yes | Yes | No | Yes | Not applicable |
| 513 | Antony 2010 Case 9 | Yes | Yes | Yes | No | Yes | Not applicable |
| 514 | Antony 2010 Case 10 | Yes | Yes | Yes | No | Yes | Not applicable |
| 515 | Antony 2010 Case 11 | Yes | Yes | Yes | No | Yes | Not applicable |
| 516 | Antony 2010 Case 12 | Yes | Yes | Yes | No | Yes | Not applicable |
| 517 | Antony 2010 Case 13 | Yes | Yes | Yes | No | No | Not applicable |
| 518 | Antony 2010 Case 14 | Yes | Yes | Yes | No | Yes | Not applicable |
| 519 | Antony 2010 Case 15 | Yes | Yes | Yes | No | Yes | Not applicable |
| 520 | Antony 2010 Case 16 | Yes | Yes | Yes | No | Yes | Not applicable |
| 521 | Antony 2010 Case 17 | Yes | Yes | Yes | No | Yes | Not applicable |
| 522 | Pandey 2010 Case 1 | Yes | Yes | Yes | Yes | Yes | Not applicable |
| 523 | Pandey 2010 Case 2 | Yes | Yes | Yes | Yes | Yes | Not applicable |
| 524 | Pandey 2010 Case 3 | Yes | Yes | Yes | Yes | Yes | Not applicable |
| 525 | Pandey 2010 Case 4 | Yes | Yes | Yes | Yes | Yes | Not applicable |
| 526 | Pandey 2010 Case 5 | Yes | No | Yes | No | Yes | Not applicable |
| 527 | Dhodapkar 2008 Case 1 | Yes | Yes | Yes | Yes | Yes | Not applicable |
| 528 | Thomas 2008 Case 1 | Yes | Yes | Yes | Yes | Yes | Not applicable |
| 529 | Mathew 1999 Case 1 | Yes | Yes | No | Yes | Yes | Not applicable |
| 530 | Mathew 1999 Case 2 | Yes | Yes | No | Yes | Yes | Not applicable |
| 531 | Mathew 1999 Case 3 | Yes | No | No | No | Yes | Not applicable |
| 532 | Mathew 1999 Case 4 | Yes | No | Yes | Yes | Yes | Not applicable |
| 533 | Mathew 1999 Case 5 | Yes | No | No | Yes | Yes | Not applicable |
| 534 | Mukhopadhyay 2008 Case 1 | Yes | No | Yes | No | Yes | Not applicable |
| 535 | Mukhopadhyay 2008 Case 2 | Yes | No | Yes | No | Yes | Not applicable |
| 536 | Mukhopadhyay 2008 Case 3 | Yes | No | Yes | No | Yes | Not applicable |
| 537 | Mukhopadhyay 2008 Case 4 | Yes | No | Yes | No | Yes | Not applicable |
| 538 | Mukhopadhyay 2008 Case 5 | Yes | No | Yes | No | No | Not applicable |
| 539 | Mukhopadhyay 2008 Case 6 | Yes | No | Yes | No | Yes | Not applicable |
| 540 | Mukhopadhyay 2008 Case 7 | Yes | No | Yes | No | No | Not applicable |
| 541 | Mukhopadhyay 2008 Case 8 | Yes | No | Yes | No | No | Not applicable |
| 542 | Mukhopadhyay 2008 Case 9 | Yes | No | Yes | No | Yes | Not applicable |
| 543 | Mukhopadhyay 2008 Case 10 | Yes | No | Yes | No | Yes | Not applicable |
| 544 | Mukhopadhyay 2008 Case 11 | Yes | No | Yes | No | Yes | Not applicable |
| 545 | Mukhopadhyay 2008 Case 12 | Yes | No | Yes | No | Yes | Not applicable |
| 546 | Mukhopadhyay 2008 Case 13 | Yes | No | Yes | No | Yes | Not applicable |
| 547 | Mukhopadhyay 2008 Case 14 | Yes | No | Yes | No | Yes | Not applicable |
| 548 | Mukhopadhyay 2008 Case 15 | Yes | No | Yes | No | Yes | Not applicable |
| 549 | Mukhopadhyay 2008 Case 16 | Yes | No | Yes | No | Yes | Not applicable |
| 550 | Mukhopadhyay 2008 Case 17 | Yes | No | Yes | No | Yes | Not applicable |
| 551 | Mukhopadhyay 2008 Case 18 | Yes | No | Yes | No | Yes | Not applicable |
| 552 | Mukhopadhyay 2008 Case 19 | Yes | No | Yes | No | Yes | Not applicable |
| 553 | Mukhopadhyay 2008 Case 20 | Yes | No | Yes | No | Yes | Not applicable |
| 554 | Mukhopadhyay 2008 Case 21 | Yes | No | Yes | No | Yes | Not applicable |
| 555 | Mukhopadhyay 2008 Case 22 | Yes | No | Yes | No | Yes | Not applicable |
| 556 | Mukhopadhyay 2008 Case 23 | Yes | No | Yes | No | Yes | Not applicable |
| 557 | Mukhopadhyay 2008 Case 24 | Yes | No | Yes | No | No | Not applicable |
| 558 | Mukhopadhyay 2008 Case 25 | Yes | No | Yes | No | No | Not applicable |
